# The Meristogram: a neglected tool for acanthocephalan systematics

**DOI:** 10.3897/BDJ.4.e7606

**Published:** 2016-02-04

**Authors:** Matthew Thomas Wayland

**Affiliations:** ‡ Department of Zoology, University of Cambridge, Cambridge, United Kingdom

**Keywords:** Echinorhynchus bothniensis, Echinorhynchus brayi, Echinorhynchus gadi, Echinorhynchus salmonis, Echinorhynchus truttae, Acanthocephala, Meristogram, proboscis, hook, morphometric, meristic, phylogeny, taxonomy, systematics, software, open-source

## Abstract

**Background:**

The hooks of the acanthocephalan proboscis exhibit serial variation in size and shape. The Meristogram was developed by Huffman and Bullock (1975) to provide a graphical representation of this positional variation in hook morphology. Initial studies demonstrated the ability of the Meristogram to discriminate species within the genera *Echinorhynchus* and *Pomphorhynchus* (Huffman and Bullock 1975, Huffman and Nickol 1978, Gleason and Huffman 1981). However, the reliability of the method for taxonomic work was questioned by Shostak et al. (1986) after they found intra-specific variation in two *Echinorhynchus* species. Uncertainty about the usefulness of the Meristogram and the absence of a readily available software implementation of the algorithm, might explain why this abstract proboscis character has yet to be adopted by acanthocephalan systematists.

**New information:**

The Meristogram algorithm was implemented in the R language and a simple graphical user interface created to facilitate ease of use (the software is freely available from https://github.com/WaylandM/meristogram). The accuracy of the algorithm's formula for calculating hook cross-sectional area was validated by data collected using a digitizing tablet. Meristograms were created from data in public respositories for eight *Echinorhynchus* taxa: *E. bothniensis*, *E. 'bothniensis'*, *E. gadi* spp. A, B and I, *E. brayi*, *E. salmonis* and *E. truttae*. In this preliminary analysis, the meristogram differentiated *E. bothniensis*, *E. brayi*, *E. gadi* sp. B, *E. salmonis* and *E. truttae* from each other, and from the remaining taxa in this study, but independent data will be required for validation. Sample sizes for *E. 'bothniensis'* and *E. gadi* spp. A and I were too small to identify diagnostic features with any degree of confidence. Meristogram differences among the sibling species of the *E. gadi* and *E. bothniensis* groups suggest that the 'intra-specfic' variation in meristogram previously reported for some *Echinorhynchus* taxa, may have actually represented morphological divergence between unrecognized cryptic species. Hierarchical clustering of taxa based on Meristogram data yielded dendrograms that were largely concordant with phylogenetic relationships inferred from DNA sequence data, indicating the presence of a strong phylogenetic signal.

## Introduction

Acanthocephalans are characterized by the presence of a retractable proboscis armed with recurved hooks which provides the means of attachment to the definitive host's gut wall. In many acanthocephalans the proboscis hooks are arranged in longitudinal rows and display positional variation in size and shape. The Meristogram was developed by Huffman and Bullock (1975) to provide a graphical representation of this serial variation in hook morphometrics. Four standardized hook parameters are plotted against longitudinal position on the proboscis; these are: blade length; blade base width; a measure of hook robustness; and an estimate of hook area. The shape of the four curves and the patterns formed through their intersection provide diagnostic characters.

Huffman and Bullock (1975) applied Meristogram analysis to collections of *Echinorhynchus gadi* Zoega in Müller, 1776 from New England and discriminated two groups: one from *Pseudopleuronectes americanus* (Walbaum, 1792) and *Limanda ferruginea* (Storer, 1839) possessing an "arched" Meristogram, and another from *Gadus morhua* L., *Pollachius virens* L., and *P. americanus* having a "looped" Meristogram. The latter group could be subdivided into two populations on the basis of differences in absolute blade length, although they possessed identical Meristograms. One population was found in *P. americanus* and the other in *G. morhua* and *P. virens*. Now recognised, all three populations can apparently be readily distinguished using conventional morphometrics, without the aid of Meristograms. Huffman and Bullock (1975) also described a distinct Meristogram for *Echinorhynchus salmonis* Müller, 1784 and demonstrated differences between the Meristograms of three species of *Pomphorhynchus* Monticelli, 1905: *P. bulbocolli* Linkins in Van Cleave, 1919, *P. sebastichthydis* Yamaguti, 1939 and *P. yamagutii* Schmidt & Hugghins, 1973. In subsequent studies, Meristogram analysis was successfully applied to the problem of discriminating *Pomphorhynchus rocci* Cordonnier & Ward, 1967 from *P. bulbocolli* (Huffman and Nickol 1978, Gleason and Huffman 1981).

Presumably anticipating the adoption of the Meristogram as a taxonomic character, Huffman and Kliever (1977) used it in their description of *Echinorhynchus canyonensis*. However, the Meristogram hasn't featured in subsequent descriptions of species in *Echinorhynchus* (Liu et al. 1981, Chandra et al. 1982, Rodjuk 1984, Parukhin 1985, Rodjuk 1986, Kovalenko 1986, Zdzitowiecki 1986, Zdzitowiecki and Valtonen 1987, Dydenko 1992, Wayland et al. 1997, Wayland et al. 1999, Smales 2012).

Shostak et al. (1986) investigated morphological variability in *E. gadi*, *E. leidyi* Van Cleave, 1924 and *E. salmonis* from 30 collection sites across northern Canada. Meristogram analysis demonstrated some intraspecific similarities and interspecific differences, but its reliability was questioned because of between-region inconsistencies in the patterns. Intraspecific variation was particularly noticeable in *E. salmonis*, with two populations displaying Meristograms which differed from each other and from another published Meristogram for *E. salmonis* (see Huffman and Bullock 1975). Such findings led Shostak et al. (1986) to reject the claim of Huffman and Nickol (1978) that the Meristogram produces a "fingerprint" that is consistent within species.

All of the previous Meristogram studies were conducted before molecular markers were in widespread use and so genetic data were not collected to demonstrate reproductive isolation between populations, detect morphologically cryptic species or resolve phylogenetic relationships between taxa. Now both hook measurements and molecular data are available for a number of *Echinorhynchus* spp. (Wayland et al. 1999, Wayland et al. 2004, Wayland et al. 2005, Wayland 2013, Wayland et al. 2015), we have the opportunity to make a more objective assessment of the Meristogram's application to acanthocephalan systematics. Key questions to be addressed are:

1. Is the Meristogram consistent with species?
2. Can the Meristogram discriminate taxa, including morphologically cryptic species?
3. Are hook patterns phylogenetically informative?

Additional aims of this study were to implement a freely available and easy to use software tool for creating Meristograms and to evaluate the accuracy of the algorithm's estimation of cross-sectional hook area.

## Material and methods

### Hook measurement data

All hook measurement data were extracted from the Dryad data repository (Wayland et al. 2013a, Wayland et al. 2013b, Wayland et al. 2013c) or the supplementary material of Wayland (2013). Importantly, the same protocol was used to measure hooks in these various studies. In brief, acanthocephalans were prepared for light microscopy by dehydration through an alcohol series followed by clearing in lactophenol. Measurements were made with aid of a digitizing tablet (KS 100, Version 3, Carl Zeiss Vision). Hook morphometric data (blade length and base width) were recorded from one longitudinal row in which all of the hooks were visible in profile using the method described by Huffman and Bullock (1975). Additionally, hook cross-sectional area was measured in samples of *E. gadi* Zoega in Müller, 1776 spp. Details of taxa sampled and their associated hook measurement data files can be found in Table 1. In the case of *E. salmonis* Müller, 1784, proboscis orientation was determined, and one dorsal row of hooks, and one ventral row was measured in each specimen.

Nuclear and mitochondrial DNA sequences have elucidated the phylogenetic relationships of six of these taxa: *E. bothniensis* Zdzitowiecki & Valtonen, 1987, *E. 'bothniensis'* (an allopatric, sibling species of *E. bothniensis*), *E. brayi* Wayland, Sommerville & Gibson, 1999, *E. gadi* sp. I (one of the sibling species of the *E. gadi* group identified by Väinölä et al. 1994 using allozymes), *E. salmonis* and the River Carron population of *E. truttae* Schrank, 1788 (Wayland et al. 2015). *E. gadi* sp. A and *E. gadi* sp. B are sympatric sibling species, discriminated using allozyme electrophoresis (Wayland et al. 2005). Their phylogenetic relationships to the other taxa are unknown, but some morphological evidence suggests that *E. gadi* sp. A and *E. gadi* sp. I might be conspecific (Wayland et al. 2005). *N.B.* in this paper the cryptic species of the *E. gadi* group are referred to using the notation of Väinölä et al. (1994) and Wayland et al. (2005); *E. gadi* spp. is used to designate the species group as a whole.

### Implementation of the Meristogram

For the original description of the Meristogram algorithm, please refer to Huffman and Bullock 1975. In brief, the Meristogram algorithm comprises the following steps:

1. Hook position is standardized to allow homologous regions of the proboscis to be compared across specimens with different numbers of hooks per row. Counted position number is multiplied by 100 and divided by n + 1, where n is the total number of hooks in the row and the constant 1 is a corrective factor for centring the data-points in graphs.
2. Two additional variables are computed for each hook: area = length × base/2 and ratio = base × 100/length.
3. Hook variables length, base, area and ratio are standardized within each row by conversion to percentages of their respective maximum value.
4. A moving average routine is used to extract the trends in positional variation for each of the hook variables. The moving average routine considers a user-defined segment of the percent-position axis for each character and moves through the data from the distal end to the proximal end of the proboscis in 1% increments. With each advance of the segment, the arithmetic means of the standardized hook variables and the percent-position value are calculated for all hooks located in the segment.
5. A moving average routine is used to extract the trends in positional variation for each of the hook variables. The moving average routine considers a user-defined segment of the percent-position axis for each character and moves through the data from the distal end to the proximal end of the proboscis in 1% increments. With each advance of the segment, the arithmetic means of the standardized hook variables and the percent-position value are calculated for all hooks located in the segment.
6. For each hook variable, each of the segment means is multiplied by 100 and divided by the largest segment mean for that hook variable, yielding a "percent-max-collection-value".
7. The percent-max-collection-values of all four hook variables are plotted against percent-position to produce the Meristogram.

The moving average routine results in a non-uniform distribution of data-points along the percent-position axis. In the current implementation of the Meristogram algorithm an optional linear interpolation procedure is introduced between steps 4 and 5. The 'approx' function in R is used to calculate hook metrics (L, B, A & R) at each integer value in the range of percent-position values yielded by the moving average routine. This linear interpolation step often improves the appearance of the Meristogram, but more importantly, by providing common percent-position coordinates it facilitates comparison of Meristograms using multivariate statistics (please see next section).

This new implementation of the Meristogram algorithm also enforces a minimum moving average segment size. It seems reasonable to argue that each proboscis in a collection should be represented in every moving average segment. The minimum moving average interval is thus defined as 100/(x+1), rounded up to the nearest integer, where n is the length of the shortest row of hooks in the collection.

**Figure 1.**
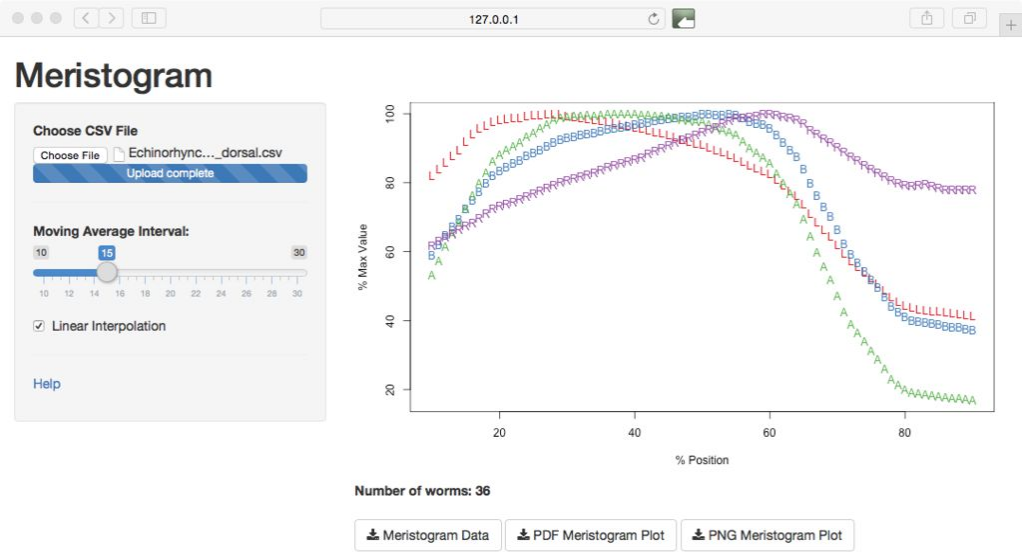
Screen-grab of the graphical user interface to the Meristogram tool. The slider control provides dynamic control of the moving average interval and linear interpolation can be toggled on/off using the checkbox. Plots and data can be downloaded using the buttons at the bottom of the screen.

The Meristogram algorithm has been implemented in R (R Core Team 2015) with a graphical user interface developed using the shiny web application framework (Chang et al. 2015). Source code is available under version 3 of the GNU General Public License from the following repository: https://github.com/WaylandM/meristogram. All Meristograms reported in this study were generated using version 1.0 of the software (DOI: 10.5281/zenodo.34267). Experienced users of R can call the functions to generate and display Meristograms from the command line. The graphical user interface (Fig. 1) enables users to interactively control the moving average segment size, toggle linear interpolation on or off and save Meristogram data and plots to file. Full instructions on using the software can be found in the documentation: https://github.com/WaylandM/meristogram/blob/master/README.md. The Meristogram software will run on any platform compatible with R, which includes Windows, Mac OS X and Linux.

### Data analysis

All data analyses were performed using the R language and environment (R Core Team 2015) and can be reproduced by running the script **data_analysis.R** provided as Suppl. material 1. The script is fully annotated and has been made available to enable readers to evaluate the methodology and verify the findings of this study.

Before considering inter-taxon differences in Meristogram, intra-taxon variation related to sex and choice of moving average interval was investigated. For each taxon, a set of Meristograms were plotted for each sex using the following moving average segment sizes: (1) minimum moving average interval (MMAI) for the taxon; (2) 1.5 × MMAI; (3) 2 × MMAI.

To assess inter-taxon variation, hook measurement data for females and males were pooled and a Meristogram was generated for each taxon using a moving average segment of 17% and linear interpolation. For comparative purposes it was important to use the same moving average segment for all Meristograms and 17% was the MMAI which could be applied to all taxa.

In addition to visual inspection of Meristograms, a more objective comparison of Meristograms was achieved with the aid of statistical pattern recognition methods. Meristogram data from all taxa were concatenated into a single matrix, where columns were hook variables at each integer percent-position and rows were taxa. Principal component analysis (PCA) was then used to reduce the number of dimensions in this data-set. Similarity of Meristograms was further assessed by hierarchical clustering of the scores for the first two principal components (accounting for most of the variation in the data-set) using the unweighted pair group method with arithmetic mean (UPGMA) algorithm and four different distance metrics (Euclidean, Manhattan, maximum and Minkowski).

## Data resources

No new data are presented in this study. However, files of hook measurement data in the appropriate format for input to the Meristogram software have been prepared and these are available as supplementary material (Suppl. materials 3, 4, 5, 6, 7, 8, 9, 10, 11, 12, 13, 14, 15, 16, 17, 18, 19). Suppl. material 2 contains measured and estimated hook cross-sectional area for *E. gadi* spp.

## Results

### Estimation of hook area

Measurements of hook cross-sectional area, obtained using a digitizing tablet, were available for *E. gadi* spp. A, B & I. These measurements were strongly correlated with area calculated by the Meristogram (Fig. 2a, b, c). Coefficients of determination (R^2^) were 0.887, 0.882 and 0.816 for *E. gadi* spp. A, B & I, respectively. The estimated hook areas were remarkably close to the measured values for all three taxa. Linear regression demonstrated that in *E. gadi* spp. A & B, the Meristogram tended to slightly over-estimate hook area (Fig. 2a, b). In *E. gadi* sp. I, the Meristogram over-estimated the area of small hooks and under-estimated the area of large hooks, however the differences were minimal (Fig. 2c).

### Meristograms

Strong evidence of sexual dimorphism was not found in the Meristograms for *E. salmonis* (Suppl. material 28) or *E. truttae* (Suppl. material 29), and sample size was too small to make a meaningful assessment of this phenomenon in the other taxa (Suppl. materials 22, 23, 24, 25, 26, 27). Thus for inter-taxon comparisons a single Meristogram was created for each taxon by pooling hook data from females and males. Variation in Meristogram pattern related to the size of the moving average segment was subtle (Suppl. materials 22, 23, 24, 25, 26, 27, 28, 29); nevertheless, for comparative purposes a 17% moving average segment (the smallest applicable to all acanthocephalans in the study) was used for all taxa.

The new software implementation of the Meristogram includes an optional linear interpolation step. Fig. 3a is the Meristogram for *E. brayi* generated using the original algorithm; data-points are not uniformly distributed along the percent-position axis. Fig. 3b is the same Meristogram smoothed using linear interpolation. The linear interpolation step doesn't alter the pattern displayed in the Meristogram, but can make it easier to visualize, especially in the case of acanthocephalans with small numbers of hooks per row, such as *E. brayi*.

Meristograms for each of the taxa in this study are presented below along with a brief description. For each taxon the meristogram characteristics which differentiate it from the other taxa in the study are identified. These differences have been termed 'Potential diagnostic features', because they have yet to be validated using independent data.

***E. brayi***

*Description*

Arched pattern with maximal values for L, B, A and R at positions 55%, 58%, 58% and 59%, respectively (Fig. 3b). Therefore, the largest and most robust hooks are expected to be found just proximal to the middle of a row. Although female and male worms exhibit the same pattern of serial variation in hook morphology (Suppl. material 24), they differ in the raw (pre-standardization) values for the four hook characters. For example, in female worms row maxima for L, B, A and R had the following ranges: 77-90µm, 24-30µm, 954-1,350µm^2^ and 30.2-35.1, respectively. In male worms the largest values of L, B, A and R were: 60-75µm, 19.5-22.5µm, 585-798µm^2^ and 29.8-35.3, respectively.

*Potential diagnostic features*

The following features of the Meristogram of *E. brayi* are unique among the taxa in this study:

1. The curves for all four hook variables converge at a percent-position of approximately 60% and all four variables have maximal values at this location.
2. On the distal third of the proboscis the order or the curves from top to bottom is length, ratio, base, area; in the other taxa the order of these hook characters is typically length, area, base, ratio.
3. The largest values of length are found in the middle of the proboscis, whereas in the other taxa maximum values of length are found towards the distal end.
4. Absence of loops formed by the intersection of curves.

***E. salmonis***

*Description*

Of the taxa in this study, only *E. salmonis* is known to display radial asymmetry in proboscis armature (Wayland et al. 2004). The Meristograms for dorsal (Fig. 4a) and ventral (Fig. 4b) hook rows are almost identical. Both display several loops, the most prominent being: a large distal loop bordered by L and B above, and R below; a proximal loop bordered above by B, and below by A and L. Maximal values of the four hook variables occurred at similar positions in both Meristograms. Dorsal hooks exhibit peak values of L, B, A and R at positions 26%, 51%, 37% and 59%, respectively; corresponding positions for ventral hooks are 27%, 59%, 37% and 64%, respectively. Therefore one would anticipate to find the longest hooks approximately a quarter of the way along a row from the distal end of the proboscis, the hooks with the greatest cross-sectional area around a third of the way along the row and the stoutest hooks just proximal to the middle of the proboscis.

In female worms the dorsal row maxima for raw L, B, A and R had the following ranges 95.7-126.7µm, 25.5-45.5µm, 1,265-2,494µm^2^ and 29.1-43.8, respectively; corresponding values for ventral rows were 99.9-129.4µm, 25.7-47.2µm, 1,410-2,678µm^2^ and 26.9-41.7, respectively. The hooks of male worms were typically smaller with dorsal row maxima for L, B, A and R of 70.1-91.3µm, 21.7-27.7µm, 720.4-1,095.0µm^2^ and 31.3-39.8, respectively; and ventral row maxima of 76.9-97.5µm, 25.2-27.7µm, 896-1,278µm^2^ and 31.4-35.5, respectively.

There is a region of very small hooks (low values of A and L) on the dorsal surface of the proximal end of the proboscis (positions > 79%), corresponding to a flattening of the tail of the Meristogram. Length and area of the last and smallest hook in dorsal rows of female worms ranged from 34.3-59.2µm and 137-477µm^2^, respectively. In male worms the final hook in a dorsal row had a length of 26.4-42.1µm and an area of 94.8-242.1µm^2^. On the ventral surface, hooks also become much smaller proximally, but not to the extent observed on the dorsal surface. The final hook in ventral rows of female worms measured 42.8-76.8µm in length and had an area of 218-782µm^2^; the corresponding hook in male worms had a length of 41-62µm and an area of 211-527µm^2^. The smallest hooks are not the weakest; the latter are found at the distal end of the proboscis. In female worms the stoutness ratio of the first hook in a row was 15.5-25.1 on the dorsal surface of the proboscis and 12.7-23.9 on the ventral. The most distal hooks of male worms had stoutness ratios of 18.3-27.8 and 17.6-30.1 for dorsal and ventral rows, respectively.

Huffman and Bullock (1975) and Shostak et al. (1986) plotted Meristograms for *E. salmonis*, but did not distinguish between dorsal and ventral hook rows; their Meristograms resemble those presented here.

*Potential diagnostic features*

1. Radial asymmetry
2. Hooks are markedly reduced in size at the proximal end of the proboscis, especially on the dorsal surface.

***E. truttae***

*Description*

The Meristograms of *E. truttae* from Drummore and the River Carron catchment display several loops; three prominent loops are shared by both collections (Fig. 5). In the 20-60% position region are two elongated loops, the upper defined by A and B, the lower by B and R. Between positions 60-80% is a loop bordered above by R and A, and below by L.

The Drummore collection has peak values of L, B, A and R at positions 40%, 72%, 70% and 77%, respectively (Fig. 5a). The longest hooks (71.1-81.4µm in females and 71.0-80.2µm in males) occur just distal to the middle of a row, and the shortest hooks (43.6-65.8µm in females and 47.7-65.3µm in males) at the proximal end of a row. The stoutest hooks (ratio values of 29.0-40.3 in females and 30.0-36.4 in males) are found in the proximal third of the row and the weakest (17.1-31.1 in females and 16.8-30.0 in males) at the distal end of the row. Hooks with maximal values of area (731-1,017µm^2^ in females and 763-988µm^2^ in males) and base (21.3-27.3µm in females and 21.6-26.6µm in males) are located around two-thirds of the way along a row from the distal end of the proboscis.

In the collection from the River Carron catchment maximal values of L, B, A and R are found at positions 33%, 64%, 43% and 75%, respectively (Fig. 5b). The longest hooks (67.6-91.0µm in females and 66.6-84.0µm in males) are found a third of the way along a row from the distal end of the proboscis, and the shortest hooks (42.6-66.2µm in females and 42.9-59.0µm in males) at the proximal end of a row. The most robust hooks (ratio values of 28.2-34.7 in females and 28.9-33.9 in males) are located in the proximal third of the row and weakest (18.0-26.0 in females and 17.1-26.7 in males) at the distal end of the row, mirroring the distribution in the Drummore collection. The hooks with greatest area (674-1,033µm^2^ in females and 626-1,096µm^2^ in males) are usually situated just distal to the middle of the row. The hooks with the broadest base (20.3-24.5µm in females and 19.2-26.1µm in males) are found two-thirds of the way along the row from the distal end of the proboscis.

*Potential diagnostic features*

*E. truttae* exhibits the least serial variation in hook size. The shortest hooks are 80% (River Carron catchment) - 82% (Drummore) of the length of the longest hooks in their respective rows. In the other taxa studied the shortest hooks are 42-71% of the length of the longest hooks. Similarly the smallest hooks of *E. truttae* have an area 62% (Drummore) - 66% (River Carron catchment) that of the largest hooks, whereas in the other taxa this value ranges from 17-51%.

***E. bothniensis***

*Description*

The curves for B, A and R follow a similar trajectory in the distal half of the Meristogram, with a particularly strong monotonic relationship between B and A (Fig. 6a). In this region a narrow loop is formed by B and R; a similar loop was observed in both *E. salmonis* and *E. truttae*. A smaller loop occurs in the 60-75% position region of the proboscis, bordered above by B and below by A and L; this latter loop is also present in the Meristogram for *E. salmonis*. Peak values for L, B, A and R are found at positions 34%, 53%, 52% and 61%, respectively. The Meristogram of *E. bothniensis* resembles the "arched pattern" Meristogram of *E. gadi* from *Pseudopleuronectes americanus* and *Limanda ferruginea* described by Huffman and Bullock (1975).

The longest hooks (57.2-66.0µm in females and 50.0-60.6µm in males) are located around a third of the way along a row from the distal end of the proboscis, and the shortest hooks (30.2-42.4µm in females and 24.7-43.3µm in males) at the proximal end of a row. The stoutest hooks (ratio values of 35.8-39.0 in females and 32.0-35.0 in males) are found just proximal to the middle of the row and the weakest (20.7-24.2 in females and 20.8-28.9 in males) at the distal end of the row. The hooks with the broadest base (18.7-22.5µm in females and 16.1-19.5µm in males) and greatest area (507-676µm^2^ in females and 393-547µm^2^ in males) are located in the middle of the row.

*Potential diagnostic features*

Apparently unique characteristics for this Meristogram are:

1. Similar curves for A, B and R in distal half of the Meristogram, with B and A almost superimposed.
2. Absence of large loops. Only two prominent loops, both of which are very narrow relative to those displayed in the Meristograms of the other taxa.

***E. 'bothniensis'***

*Description*

The Meristogram of *E. 'bothniensis'* (Fig. 6b) should be interpreted with particular caution, because it is based on hook data from just two female worms. Inclusion of additional samples would likely smooth the curves of this Meristogram. Maximal values of L, B, A and R occur at positions: 31%, 74%, 33% and 74%. It features four prominent loops, one in the distal half and three in the proximal half. The longest (63.5-67.6µm) and largest (596-747µm^2^) hooks are situated a third of the way along the row from the distal end of the proboscis, and the shortest (33.0-39.3µm) and smallest (135-187µm^2^) hooks at the proximal end of the row. The hooks with the broadest base (21.9-22.7µm) are also the stoutest (ratio values of 40.7-40.8), and are located in the proximal third of the row. The weakest hooks (ratio values of 19.0-22.3) are located at the distal end of the row.

*Potential diagnostic features*

The Meristogram for *E. 'bothniensis'* was created using data from just two specimens; additional data may well change the appearance of the Meristogram. For this reason diagnostic characters have not been proposed for this taxon.

***E. gadi* sp. A**

*Description*

Maximal values of L, B, A and R occur at positions: 27%, 67%, 57% and 67% (Fig. 6c). In the region 45-75% position, three prominent loops are fomed by intersection of the four curves: the most distal defined by A, L and B; the middle loop bordered by B, L and R; the most distal loop composed of R, A and L. The longest hooks (66.1-72.6µm in females and 57.4-67.3µm in males) are located a quarter of the way along a row from the distal end of the proboscis, and the shortest hooks (40.9-49.1µm in females and 36.8-44.1µm in males) at the proximal end of the row. The hooks with the broadest base (26.0-32.1µm in females and 23.8-26.0µm in males) are also the stoutest (ratio values of 41.4-50.7 in females and 44.4-46.5 in males), and are situated two thirds of the way along a row from the distal end of the proboscis. The weakest hooks (ratio values of 19.1-29.3 in females and 26.1-27.2 in males) are found at the proximal end of the proboscis. Hooks with the greatest area (747-1,072µm^2^ in females and 628-761µm^2^ in males) are found just proximal to the middle of a row.

*Potential diagnostic features*

*E. gadi* sp. A is one of only three taxa in this study with a Meristogram in which none of the curves intersect at the distal end of the proboscis; the others are *E. gadi* sp. I and *E. brayi*. Differences in the shape of the curve for A might separate *E. gadi* sp. A from *E. gadi* sp. I, however given the small sample sizes for these two collections of worms, this diagnostic may not be robust. Moreover, there is tentative evidence that these two taxa are conspecific (Wayland et al. 2005). Meristogram characteristics distinguising *E. brayi* from all other taxa in this study are described above.

***E. gadi*** **sp. B**

*Description*

The Meristogram of *E. gadi* sp. B possesses three prominent loops: a distal loop bordered above by B and L, and below by R; a loop mid-way along the proboscis bordered above by B, and below by A and R; a proximal loop bordered above by R and B, and below by A and L (Fig. 6d). Maximal values for L, B, A and R are at positions 25%, 42%, 35% and 74%, respectively. The longest hooks (47.2-56.9µm in females and 45.7-54.0µm in males) are located a quarter of the way along a row from the distal end of the proboscis, and the shortest hooks (31.0-48.8µm in females and 19.9-30.5µm in males) at the proximal end of the row, a similar profile to *E. gadi* sp. A. The hooks with the broadest base (17.7-20.4µm in females and 17.3-18.6µm in males) are situated just distal to the middle of the row and the largest hooks (421-528µm^2^ in females and 400-502µm^2^ in males) are found a third of the way along the row from the distal end of the proboscis. The stoutest hooks (ratio values of 41.0-52.1 in females and 38.9-43.3 in males) occur in the proximal third of the row.

The Meristograms of all of the *E. gadi* spp. in this study, broadly display what Huffman and Bullock (1975) describe as a "looped pattern". However, the Meristogram of *E. gadi* sp. B appears to be closest to the exemplar of the "looped pattern" depicted in Fig. 11 of Huffman and Bullock (1975).

*Potential diagnostic features*

*E. gadi* sp. B is the only taxon in this study in which the hook variable B peaks on the distal half of the proboscis.

***E. gadi* sp. I**

*Description*

In the 45-80% position region of the Meristogram are three obvious loops (Fig. 6e). The loop situated mid-way along the Meristogram, and bordered by B, A and R, is also present in the Meristograms for *E. gadi* sp. B, *E. 'bothniensis'* and *E. salmonis*. Peak values of L, B, A and R occur at positions: 18%, 68%, 24% and 69%. The longest hooks (66.2-74.2µm in females and 61.6µm in the only male specimen) are situated in the distal quarter of a row and the shortest hooks (35.9-51.1µm in females and 43.8µm in the male) at the proximal end of the row. The largest hooks (592-827µm^2^ in females and 545µm^2^ in the male) are situated in the distal third of the row. As in *E. 'bothniensis'* and *E. gadi* sp. A, the hooks with the broadest base (20.9-24.2µm in females and 21.9µm in the male) are also the stoutest (ratio values of 36.9-47.3 in females and 45.9 in the male). In *E. gadi* sp. I, these most robust hooks are located two thirds of the way along a row from the distal end of the proboscis. The weakest hooks (ratio values of 20.5-26.2 in females and 21.8 in the male) are found at the distal end of a row.

*Potential diagnostic features*

Meristogram characteristics which may differentiate *E. gadi* sp. I and A from the other taxa in this study have been described in the section on *E. gadi* sp. A (above).

### Clustering taxa on Meristogram similarity

Visual inspection of Meristograms reveals some similarities and differences between taxa. For a more objective comparison, statistical pattern recognition was used to measure Meristogram divergence between taxa. In this analysis, a single Meristogram was generated for *E. salmonis* by pooling hook measurement data from dorsal and ventral rows of hooks; the hook measurement data from the other taxa were assumed to comprise a mixture of dorsal and ventral rows of hooks. The two collections of *E. truttae* were treated as separate entities, because of the divergence observed between their Meristograms (Fig. 5).

Principal component analysis (PCA) revealed the major sources of variation in the Meristograms. Fig. 7a is a scatterplot of the scores for the first two principal components (PCs), which between them account for 83% of the variation in the data. Similar scores for these two PCs, indicate relatively little divergence in hook patterns between *E. bothniensis*, *E. 'bothniensis'*, and *E. gadi* spp. A and B. Conversely a large negative score for PC1 demonstrates that *E. salmonis* has diverged markedly from the other taxa in hook pattern. A more modest positive score for PC1, separates *E. truttae* from its congeners. The loadings plot for the first two PCs (Fig. 7b) shows that hook area in the 69-85% position region of the proboscis is driving the separation of taxa in PC1; the proximal hooks of *E. salmonis* being proportionally much smaller than those of the other taxa, whereas those of *E. truttae* are a little larger. *E. brayi* shows strong divergence from the other taxa in PC2; *E. salmonis* and *E. gadi* sp. I display some separation from their congeners in this axis. Hook area in the 15-32% position region of the proboscis is driving the separation of taxa in PC2.

Hierarchical clustering of the scores for the first two PCs depicts relationships between Meristograms as a dendrogram (Fig. 8). Three distance metrics (Euclidean, Manhattan and Minkowski) yielded identical dendrograms (Fig. 8a, b, d). The dendrogram created using the maximum distance metric was almost identical to the other three dendrograms, differing only in the depiction of the relationship between *E. salmonis* and *E. brayi*. Concordance between dendrograms created using different distance metrics suggests that the clusters found are robust.

## Discussion

### Intraspecific variation

The differences in Meristogram between the central and southwest Scotland collections of *E. truttae* suggest intraspecific, geographical variation in hook patterns. However, given the existence of cryptic species in *Echinorhynchus* (Väinölä et al. 1994), the alternative explanation of interspecific variation should also be considered. Despite this subtle difference in Meristogram, the two populations of *E. truttae* displayed a much stronger affinity to each other, than to any of the other taxa in all cluster analyses (Fig. 8). Interestingly, Proboscis Profiler, a tool inspired by the Meristogram and designed to detect heterogeneity in collections of acanthocephalans, did not classify these two collections of *E. truttae* as distinct morphotypes (Wayland 2010).

### Discrimination of taxa

None of the eight *Echinorhynchus* taxa in this study appear to share identical Meristograms, based on the available hook measurement data. However, sample sizes were small (six or fewer specimens for *E. 'bothniensis'* and *E. gadi* spp. A and I) and so very unlikely to encompass the range of morphological variation potentially displayed by these taxa. Such variation might render some of the proposed diagnostics ineffectual, particularly for the discrimination of the closely related *E. truttae*, *E. gadi* spp, *E. bothniensis* and *E. 'bothniensis'*.

Not surprisingly, Meristogram analysis appeared to be most effective at discriminating taxa for which conventional characters are diagnostic, such as *E. brayi*, *E. salmonis* and *E. truttae*; these are also the taxa showing the greatest genetic divergence (Wayland et al. 2015). Differentiation of the cryptic sibling species was more of a challenge, in part because of the small sample sizes. Nevertheless, some observations suggest diagnostic characters which could be evaluated with additional data. For example, the Meristogram of *E. bothniensis* was arched, whereas that of *E. 'bothniensis'*, was looped. If this difference is real and not an artifact of small sample size (n=10 and n=2, respectively), then this represents the first morphological character to separate these allopatric sibling species. A previous attempt to discriminate these taxa using conventional morphometrics or the Proboscis Profiler tool was unsuccessful (Wayland 2013). *E. gadi* spp. A and B have been discriminated morphologically on the basis of absolute differences in hook size in the 10-20% position region of the proboscis (Wayland et al. 2005). Meristogram analysis suggests that an additional diagnostic character could be the location of the hooks with the broadest base, which is proximal in *E. gadi* sp. A and distal in *E. gadi* sp. B.

Morphology and ecology suggest that the NE Atlantic *E. brayi* is closely related to *E. canyonensis* from the Monterey Submarine Canyon in the Pacific (Wayland et al. 1999). The two species have similar hook formulae and are anatomically almost identical, differing in only two conventional characters: the length of the lemnisci relative to the proboscis receptacle and shelled acanthor length. Both species are parasites of deep-sea zoarcid fishes. When Huffman and Kliever (1977) described *E. canyonensis*, they were aware that “many helminth populations cannot be adequately characterized with line drawings and a list of morphometrics, especially in this genus [*Echinorhynchus*]” and so included a Meristogram as an additional diagnostic character in the hope that it would “be useful in the future”. The Meristograms of *E. brayi* and *E. canyonensis* are similar in that they both show an arched pattern and the four curves intersect at around the 60% position (compare Fig. 3 with Fig. 5 of Huffman and Kliever 1977). However, there are key differences in the order of the four curves. Distally the order of the curves from top to bottom is L, A, B, R in *E. canyonensis*, whereas in *E. brayi* it is L, R, B, A. Diagnostic differences are also apparent in the proximal region, for example at the 80% position the order of the curves from top to bottom is L, R, B, A in *E. canyonensis* and R, L, B, A in *E. brayi*. It is important to note that the published Meristogram for *E. canyonensis* was created with a moving average interval of 19%; the diagnostic differences can be observed in *E. brayi* Meristograms generated using moving average intervals in the range 17-26% (Fig. 3, Suppl. material 24).

### Phylogenetic signal

**Figure 9.**
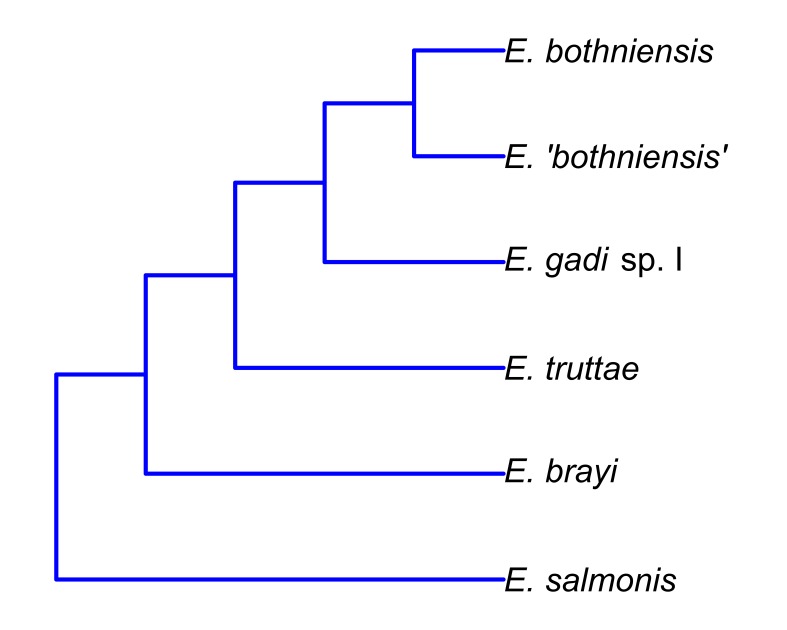
Phylogenetic relationships of six of the *Echinorhynchus* taxa in this study inferred from nuclear ribosomal and mitochondrial DNA sequences (Wayland et al. 2015). *E. gadi* spp. A and B are not shown on the above tree, because sequence data are not available for these taxa.

Hierarchical clustering shows relationships between taxa based on overall similarity (Fig. 8). This phenetic method makes no distinction between plesiomorphies and apomorphies, and therefore a dendrogram it produces may have a different topology to the actual phylogenetic tree. Fig. 9 shows the phylogenetic relationships of six of the taxa in this study, as inferred from nuclear and mitochondrial DNA sequences (Wayland et al. 2015). Sequence data are not available for *E. gadi* spp. A and B. Remarkably, hierarchical clustering of Meristogram data using the maximum distance metric (Fig. 8c) yielded a dendrogram depicting relationships between the taxa which are in perfect agreement with the molecular phylogeny. Dendrograms created using three other distance metrics (Fig. 8a, b, d) clustered *E. brayi* and *E. salmonis* together, but were otherwise congruent with the molecular phylogeny.

Concordance between the dendrograms and the molecular phylogeny is evidence of a strong phylogenetic signal in the Meristogram. This raises the prospect of using Meristogram characteristics to define monophyletic groups within *Echinorhynchus* and thus facilitate a revision of this large and expanding genus. However, distillation of discrete character states suitable for taxonomy, from the continuous variables that comprise the Meristogram, is likely to be a significant challenge.

### Practical considerations

The formula used by the Meristogram for estimating hook cross-sectional area has been shown to be accurate. However, since hook metrics are standardized, estimates of hook area are only required to be proportional to the actual area. Although no evidence of sexual dimorphism was found in this study, users of the Meristogram should be alert to this possible source of intra-specific variation. In *E. truttae* females tend to have slightly larger hooks than males (Wayland 2013), but this difference is eliminated by the standardization procedures in the Meristogram algorithm. The size of conventional taxonomic characters in the Acanthocephala has been shown to vary with age, sex, species of definitive host and geography (Amin and Redlin 1980, Shostak et al. 1986). Further work is required to determine if these factors influence the appearance of the Meristogram.

Meristograms have been shown to be sensitive to the choice of moving average interval and so in comparative studies it is important to use the same value of this parameter for all hook collections. The systematic homogeneity of a sample of acanthocephalans should be assessed before applying the Meristogram. Molecular markers are the first choice of tool for detecting cryptic species, but when they cannot be applied, the Proboscis Profiler (Wayland 2010) may detect distinct morphotypes in the sample, using the same hook data collected for the Meristogram. Proboscis Profiler was inspired by the Meristogram algorithm and complements it by providing statistical pattern recognition techniques to detect morphological heterogeneity in collections of acanthocephalans.

Users of the Meristogram are encouraged to share their hook measurement data, either by including them as supplementary files to publications or submitting them to public repositories (e.g. Dryad, Zenodo, etc.). In addition to facilitating further comparative analyses, these datasets are potentially useful in the testing and refinement of the Meristogram algorithm.

### Final comments

Having demonstrated the utility of the Meristogram in acanthocephalan systematics, the author is keen to facilitate its use. Free software is provided for systematists who would like to apply the technique themselves. Additionally, the author would welcome the opportunity to analyse hook morphometric data for other acanthocephalan workers as part of a collaborative project.

The Meristogram is not limited to the analysis of the hooks of the acanthocephalan proboscis. It can potentially be applied to any organism in which there are patterns in the longitudinal variation of serially homologous structures, *e.g.* "the taxonomic diagnosis of the vegetative characters of plants when fruiting structures are not available; species-level diagnosis of feather structures in fragments encountered in gut contents (or poacher's clothing), etc." (Professor David Huffman, personal communication, July 7, 2010). The freely available source code for the current implementation of the Meristogram should provide a helpful starting point for anyone who would like to adapt the method for novel applications.

## Supplementary Material

Supplementary material 1 Data analysis script Data type: R language script
Brief description: The commands in this script will perform all of the data analyses described in this paper and generate all of the figures. The script is included here to enable readers to verify the findings of this study.
File: oo_73745.R Matthew T Wayland

Supplementary material 2 Area of hooks from Echinorhynchus gadi spp. Data type: morphometric
Brief description: Comma separated value file with the following nine columns: species, sex, specimen, hook, position, length, base, measured_area and estimated_area.
species - species of the *E. gadi* group (A, B or I)
sex - sex of acanthocephalan
hook - numerical position of hook in longitudinal row as counted from the distal end of the proboscis
length - length of hook blade (micrometres)
base - width of hook base (micrometres)
measured_area - cross-sectional area (square micrometres) of a hook viewed in profile and measured using a digitising tablet
estimated_area - cross-sectional area (square micrometres) of hook estimated using the formula: area = length x base / 2
Source: http://dx.doi.org/10.5061/dryad.hd5c7
File: oo_63675.csv Matthew T Wayland

Supplementary material 3 Hook measurement data from female Echinorhynchus bothniensis Data type: morphometric
Brief description: The file is a comma separated value (CSV) format suitable for input to the Meristogram software. The file has four columns: specimen, hook, length, base.
specimen - unique identifier for the specimen
hook - numerical position of hook in longitudinal row as counted from the distal end of the proboscis
length - length of hook blade (micrometres)
base - width of hook base (micrometres)
Source: Wayland (2013)
File: oo_63705.csv Matthew T Wayland

Supplementary material 4 Hook measurement data from male Echinorhynchus bothniensis Data type: morphometric
Brief description: The file is a comma separated value (CSV) format suitable for input to the Meristogram software. The file has four columns: specimen, hook, length, base.
specimen - unique identifier for the specimen
hook - numerical position of hook in longitudinal row as counted from the distal end of the proboscis
length - length of hook blade (micrometres)
base - width of hook base (micrometres)
Source: Wayland (2013)
File: oo_63706.csv Matthew T Wayland

Supplementary material 5 Hook measurement data from female Echinorhynchus 'bothniensis' Data type: morphometric
Brief description: The file is a comma separated value (CSV) format suitable for input to the Meristogram software. The file has four columns: specimen, hook, length, base.
specimen - unique identifier for the specimen
hook - numerical position of hook in longitudinal row as counted from the distal end of the proboscis
length - length of hook blade (micrometres)
base - width of hook base (micrometres)
Source: Wayland (2013)
File: oo_63707.csv Matthew T Wayland

Supplementary material 6 Hook measurement data from female Echinorhynchus brayi Data type: morphometric
Brief description: The file is a comma separated value (CSV) format suitable for input to the Meristogram software. The file has four columns: specimen, hook, length, base.
specimen - unique identifier for the specimen
hook - numerical position of hook in longitudinal row as counted from the distal end of the proboscis
length - length of hook blade (micrometres)
base - width of hook base (micrometres)
Source: http://dx.doi.org/10.5061/dryad.62285
File: oo_63694.csv Matthew T Wayland

Supplementary material 7 Hook measurement data from male Echinorhynchus brayi Data type: morphometric
Brief description: The file is a comma separated value (CSV) format suitable for input to the Meristogram software. The file has four columns: specimen, hook, length, base.
specimen - unique identifier for the specimen
hook - numerical position of hook in longitudinal row as counted from the distal end of the proboscis
length - length of hook blade (micrometres)
base - width of hook base (micrometres)
Source: http://dx.doi.org/10.5061/dryad.62285
File: oo_63695.csv Matthew T Wayland

Supplementary material 8 Hook measurement data from female Echinorhynchus gadi sp. A Data type: morphometric
Brief description: The file is a comma separated value (CSV) format suitable for input to the Meristogram software. The file has four columns: specimen, hook, length, base.
specimen - unique identifier for the specimen
hook - numerical position of hook in longitudinal row as counted from the distal end of the proboscis
length - length of hook blade (micrometres)
base - width of hook base (micrometres)
Source: http://dx.doi.org/10.5061/dryad.hd5c7
File: oo_63710.csv Matthew T Wayland

Supplementary material 9 Hook measurement data from male Echinorhynchus gadi sp. A Data type: morphometric
Brief description: The file is a comma separated value (CSV) format suitable for input to the Meristogram software. The file has four columns: specimen, hook, length, base.
specimen - unique identifier for the specimen
hook - numerical position of hook in longitudinal row as counted from the distal end of the proboscis
length - length of hook blade (micrometres)
base - width of hook base (micrometres)
Source: http://dx.doi.org/10.5061/dryad.hd5c7
File: oo_63711.csv Matthew T Wayland

Supplementary material 10 Hook measurement data from female Echinorhynchus gadi sp. B Data type: morphometric
Brief description: The file is a comma separated value (CSV) format suitable for input to the Meristogram software. The file has four columns: specimen, hook, length, base.
specimen - unique identifier for the specimen
hook - numerical position of hook in longitudinal row as counted from the distal end of the proboscis
length - length of hook blade (micrometres)
base - width of hook base (micrometres)
Source: http://dx.doi.org/10.5061/dryad.hd5c7
File: oo_63712.csv Matthew T Wayland

Supplementary material 11 Hook measurement data from male Echinorhynchus gadi sp. B Data type: morphometric
Brief description: The file is a comma separated value (CSV) format suitable for input to the Meristogram software. The file has four columns: specimen, hook, length, base.
specimen - unique identifier for the specimen
hook - numerical position of hook in longitudinal row as counted from the distal end of the proboscis
length - length of hook blade (micrometres)
base - width of hook base (micrometres)
Source: http://dx.doi.org/10.5061/dryad.hd5c7
File: oo_63713.csv Matthew T Wayland

Supplementary material 12 Hook measurement data from female Echinorhynchus gadi sp. I Data type: morphometric
Brief description: The file is a comma separated value (CSV) format suitable for input to the Meristogram software. The file has four columns: specimen, hook, length, base.
specimen - unique identifier for the specimen
hook - numerical position of hook in longitudinal row as counted from the distal end of the proboscis
length - length of hook blade (micrometres)
base - width of hook base (micrometres)
Source: http://dx.doi.org/10.5061/dryad.hd5c7
File: oo_63714.csv Matthew T Wayland

Supplementary material 13 Hook measurement data from male Echinorhynchus gadi sp. I Data type: morphometric
Brief description: The file is a comma separated value (CSV) format suitable for input to the Meristogram software. The file has four columns: specimen, hook, length, base.
specimen - unique identifier for the specimen
hook - numerical position of hook in longitudinal row as counted from the distal end of the proboscis
length - length of hook blade (micrometres)
base - width of hook base (micrometres)
Source: http://dx.doi.org/10.5061/dryad.hd5c7
File: oo_63715.csv Matthew T Wayland

Supplementary material 14 Hook measurement data from dorsal rows of female Echinorhynchus salmonis Data type: morphometric
Brief description: The file is a comma separated value (CSV) format suitable for input to the Meristogram software. The file has four columns: specimen, hook, length, base.
specimen - unique identifier for the specimen
hook - numerical position of hook in longitudinal row as counted from the distal end of the proboscis
length - length of hook blade (micrometres)
base - width of hook base (micrometres)
Source: http://dx.doi.org/10.5061/dryad.v21k1
File: oo_63696.csv Matthew T Wayland

Supplementary material 15 Hook measurement data from ventral rows of female Echinorhynchus salmonis Data type: morphometric
Brief description: The file is a comma separated value (CSV) format suitable for input to the Meristogram software. The file has four columns: specimen, hook, length, base.
specimen - unique identifier for the specimen
hook - numerical position of hook in longitudinal row as counted from the distal end of the proboscis
length - length of hook blade (micrometres)
base - width of hook base (micrometres)
Source: http://dx.doi.org/10.5061/dryad.v21k1
File: oo_63699.csv Matthew T Wayland

Supplementary material 16 Hook measurement data from dorsal rows of male Echinorhynchus salmonis Data type: morphometric
Brief description: The file is a comma separated value (CSV) format suitable for input to the Meristogram software. The file has four columns: specimen, hook, length, base.
specimen - unique identifier for the specimen
hook - numerical position of hook in longitudinal row as counted from the distal end of the proboscis
length - length of hook blade (micrometres)
base - width of hook base (micrometres)
Source: http://dx.doi.org/10.5061/dryad.v21k1
File: oo_63701.csv Matthew T Wayland

Supplementary material 17 Hook measurement data from ventral rows of male Echinorhynchus salmonis Data type: morphometric
Brief description: The file is a comma separated value (CSV) format suitable for input to the Meristogram software. The file has four columns: specimen, hook, length, base.
specimen - unique identifier for the specimen
hook - numerical position of hook in longitudinal row as counted from the distal end of the proboscis
length - length of hook blade (micrometres)
base - width of hook base (micrometres)
Source: http://dx.doi.org/10.5061/dryad.v21k1
File: oo_63703.csv Matthew T Wayland

Supplementary material 18 Hook measurement data from female Echinorhynchus truttae from Drummore, southwest Scotland Data type: morphometric
Brief description: The file is a comma separated value (CSV) format suitable for input to the Meristogram software. The file has four columns: specimen, hook, length, base.
specimen - unique identifier for the specimen
hook - numerical position of hook in longitudinal row as counted from the distal end of the proboscis
length - length of hook blade (micrometres)
base - width of hook base (micrometres)
Source: Wayland (2013)
File: oo_68461.csv Matthew T Wayland

Supplementary material 19 Hook measurement data from male Echinorhynchus truttae from Drummore, southwest Scotland Data type: morphometric
Brief description: The file is a comma separated value (CSV) format suitable for input to the Meristogram software. The file has four columns: specimen, hook, length, base.
specimen - unique identifier for the specimen
hook - numerical position of hook in longitudinal row as counted from the distal end of the proboscis
length - length of hook blade (micrometres)
base - width of hook base (micrometres)
Source: Wayland (2013)
File: oo_68463.csv Matthew T Wayland

Supplementary material 20 Hook measurement data from female Echinorhynchus truttae from the River Carron catchment, central Scotland Data type: morphometric
Brief description: The file is a comma separated value (CSV) format suitable for input to the Meristogram software. The file has four columns: specimen, hook, length, base.
specimen - unique identifier for the specimen
hook - numerical position of hook in longitudinal row as counted from the distal end of the proboscis
length - length of the hook blade (micrometres)
base - width of the hook base (micrometres)
Source: Wayland (2013)
File: oo_68464.csv Matthew T Wayland

Supplementary material 21 Hook measurement data from male Echinorhynchus truttae from the River Carron catchment, central Scotland Data type: morphometric
Brief description: The file is a comma separated value (CSV) format suitable for input to the Meristogram software. The file has four columns: specimen, hook, length, base.
specimen - unique identifier for the specimen
hook - numerical position of hook in longitudinal row as counted from the distal end of the proboscis
length - length of the hook blade (micrometres)
base - width of the hook base (micrometres)
Source: Wayland (2013)
File: oo_68466.csv Matthew T Wayland

Supplementary material 22 Meristograms for female and male Echinorhynchus bothniensis Data type: morphological
Brief description: Meristograms for five female and five male specimens of *Echinorhynchus bothniensis*. For each sex Meristograms were generated with the following moving average intervals: 9, 14 and 18%. These intervals correspond to the minimum moving average interval (MMAI) for this collection of worms, 1.5 x MMAI and 2.0 x MMAI, respectively.
File: oo_64487.pdf Matthew T Wayland

Supplementary material 23 Meristograms for female Echinorhynchus 'bothniensis' Data type: morphology
Brief description: Meristograms for two female specimens of *Echinorhynchus brayi*, generated with the following moving average intervals: 8, 12 and 16%. These intervals correspond to the minimum moving average interval (MMAI) for this collection of worms, 1.5 x MMAI and 2.0 x MMAI, respectively.
File: oo_64488.pdf Matthew T Wayland

Supplementary material 24 Meristograms for female and male Echinorhynchus brayi Data type: morphological
Brief description: Meristograms for four female and seven male specimens of *Echinorhynchus brayi*. For each sex Meristograms were generated with the following moving average intervals: 17, 26 and 34%. These intervals correspond to the minimum moving average interval (MMAI) for this collection of worms, 1.5 x MMAI and 2.0 x MMAI, respectively.
File: oo_64449.pdf Matthew T Wayland

Supplementary material 25 Meristograms for female and male Echinorhynchus gadi sp. A Data type: morphological
Brief description: Meristograms for four female and two male specimens of *Echinorhynchus gadi* sp. A. For each sex Meristograms were generated with the following moving average intervals: 9, 14 and 18%. These intervals correspond to the minimum moving average interval (MMAI) for this collection of worms, 1.5 x MMAI and 2.0 x MMAI, respectively.
File: oo_64463.pdf Matthew T Wayland

Supplementary material 26 Meristograms for female and male Echinorhynchus gadi sp. B Data type: morphological
Brief description: Meristograms for four female and four male specimens of *Echinorhynchus gadi* sp. B. For each sex Meristograms were generated with the following moving average intervals: 8, 12 and 16%. These intervals correspond to the minimum moving average interval (MMAI) for this collection of worms, 1.5 x MMAI and 2.0 x MMAI, respectively.
File: oo_64464.pdf Matthew T Wayland

Supplementary material 27 Meristograms for female and male Echinorhynchus gadi sp. I Data type: morphological
Brief description: Meristograms for five female and one male specimens of *Echinorhynchus gadi* sp. I. For each sex Meristograms were generated with the following moving average intervals: 8, 12 and 16%. These intervals correspond to the minimum moving average interval (MMAI) for this collection of worms, 1.5 x MMAI and 2.0 x MMAI, respectively.
File: oo_64466.pdf Matthew T Wayland

Supplementary material 28 Meristograms for female and male Echinorhynchus salmonis Data type: morphological
Brief description: Meristograms for 36 female and 6 male specimens of *Echinorhynchus salmonis*. For each sex and proboscis surface (dorsal and ventral) Meristograms were generated with the following moving average intervals: 10, 15 and 20%. These intervals correspond to the minimum moving average interval (MMAI) for this collection of worms, 1.5 x MMAI and 2.0 x MMAI, respectively.
File: oo_64457.pdf Matthew T Wayland

Supplementary material 29 Meristograms for female and male Echinorhynchus truttae Data type: morphological
Brief description: Meristograms for *Echinorhynchus truttae* from two different geographical locations: Drummore, southwest Scotland (35 females and 19 males) and the River Carron catchment, central Scotland (11 females and 7 males). For each sex and locality Meristograms were generated with the following moving average intervals: 10, 15 and 20%. These intervals correspond to the minimum moving average interval (MMAI) for this collection of worms, 1.5 x MMAI and 2.0 x MMAI, respectively.
File: oo_68417.pdf Matthew T Wayland

## Figures and Tables

**Figure 2a.**
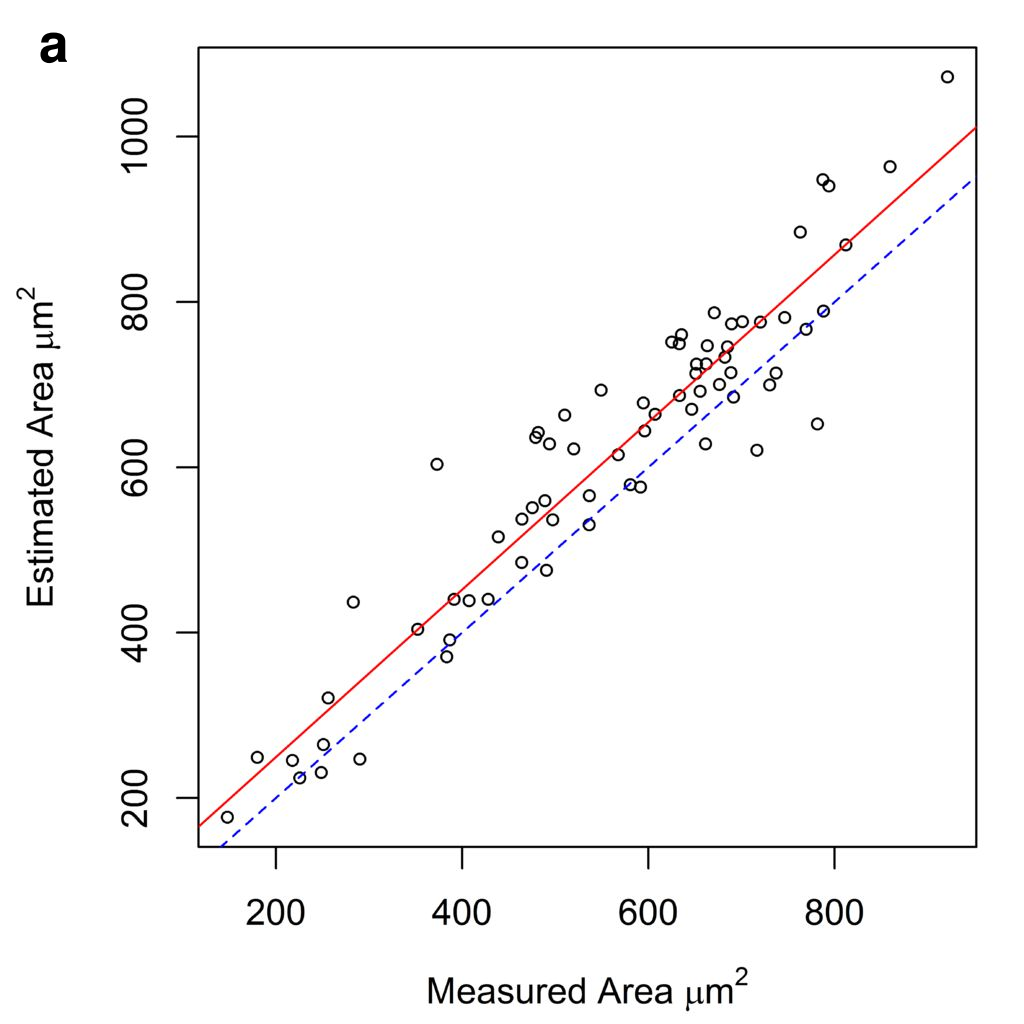
***E. gadi* sp. A.** 70 hooks from six worms (four females and two males). Equation of regression line: estimated = 1.013 x measured + 46.898. Coefficient of determination (R^2^) = 0.887.

**Figure 2b.**
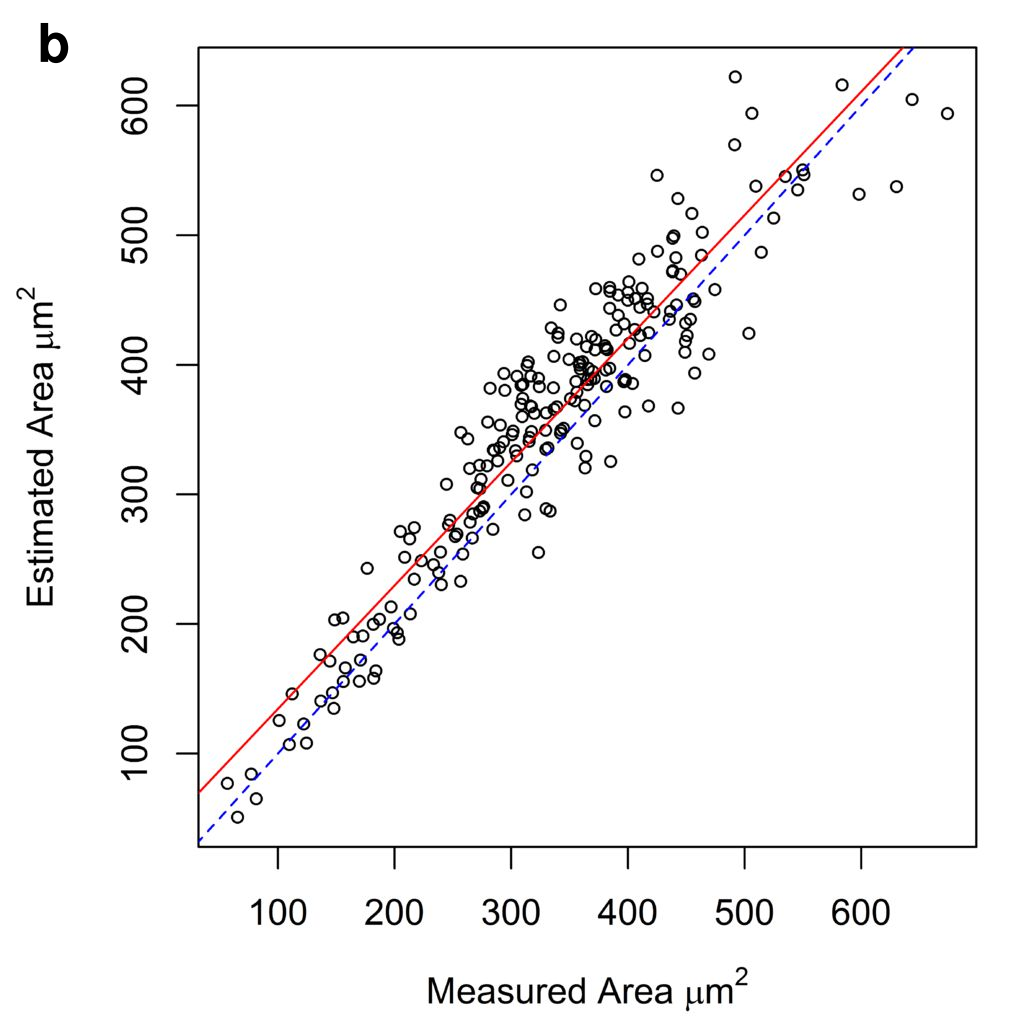
***E. gadi* sp. B.** 220 hooks from 18 worms (11 females and seven males). Equation of regression line: estimated = 0.953 x measured + 39.178. R^2^ = 0.882.

**Figure 2c.**
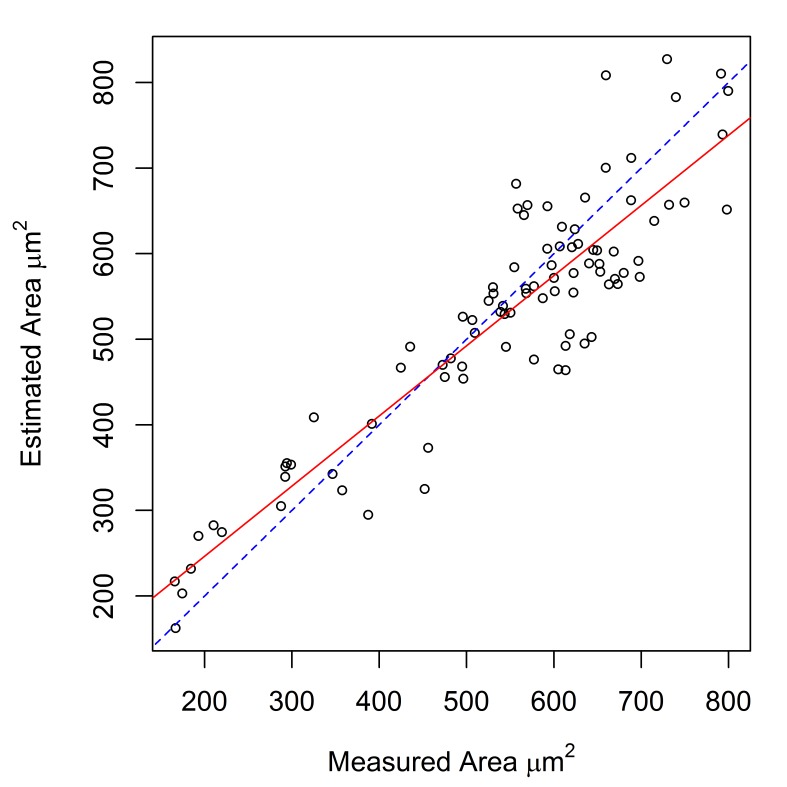
***E. gadi* sp. I.** 91 hooks from seven worms (six females and one male). Equation of regression line: estimated = 0.819 x measured + 82.640. R^2^ = 0.816.

**Figure 3a.**
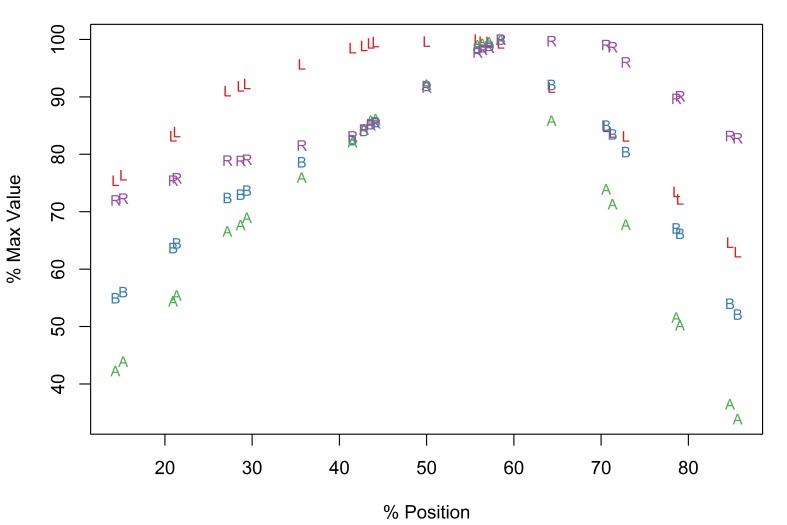
Meristogram without linear interpolation.

**Figure 3b.**
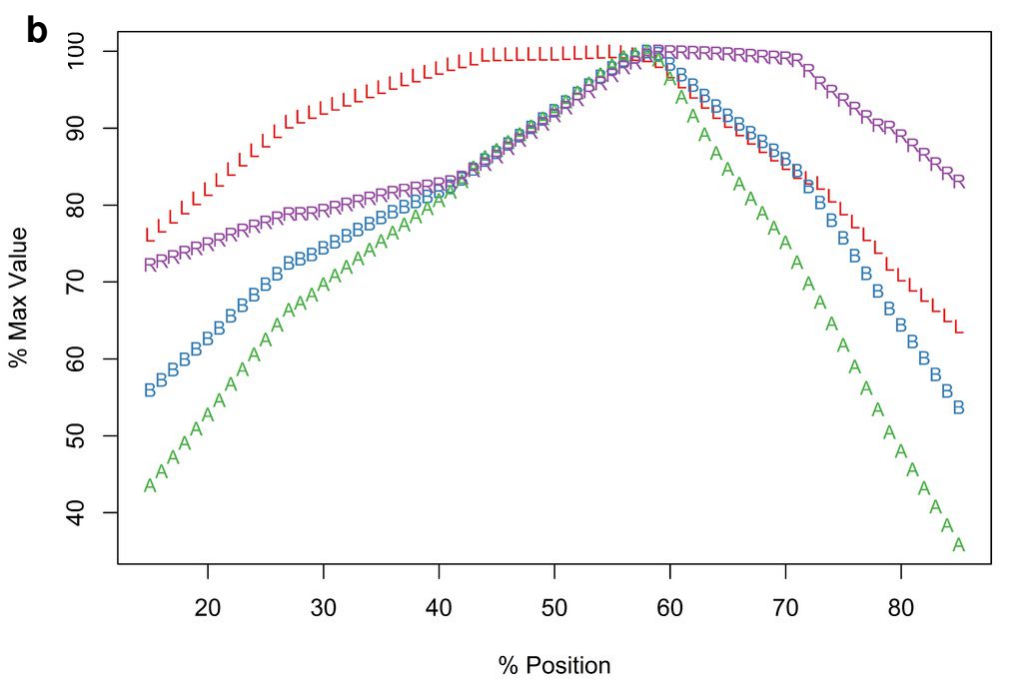
Meristogram with linear interpolation.

**Figure 4a.**
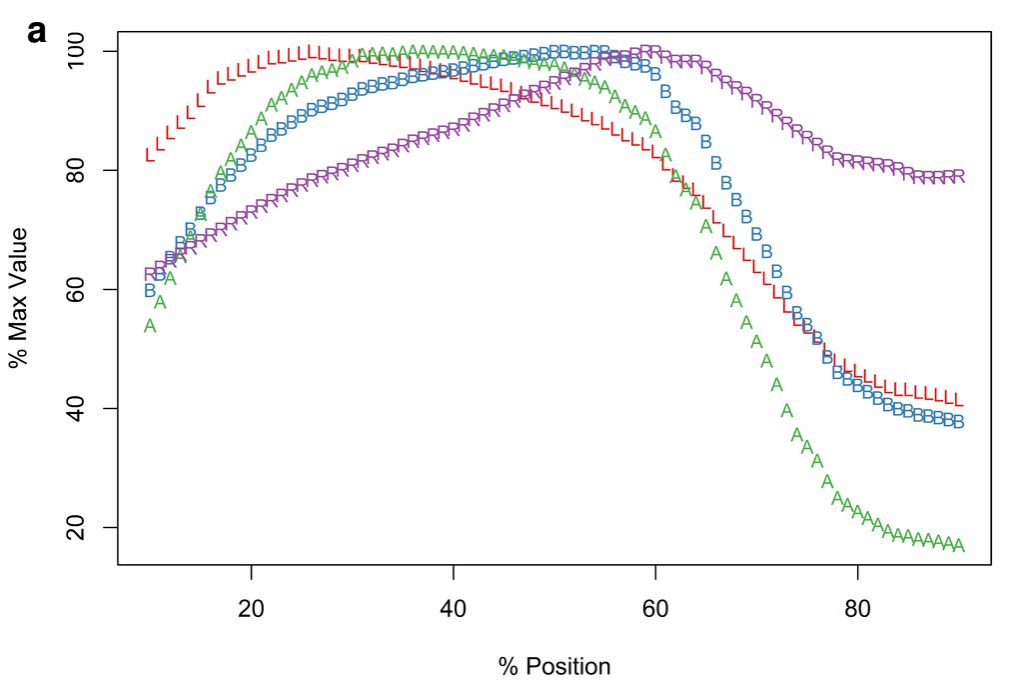
Dorsal

**Figure 4b.**
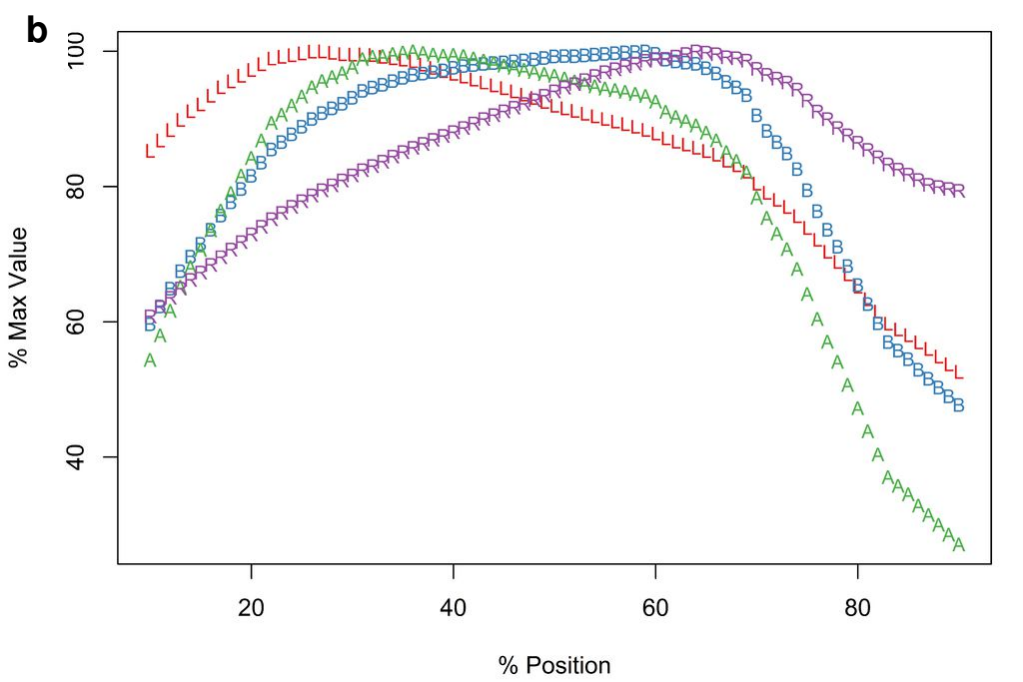
Ventral

**Figure 5a.**
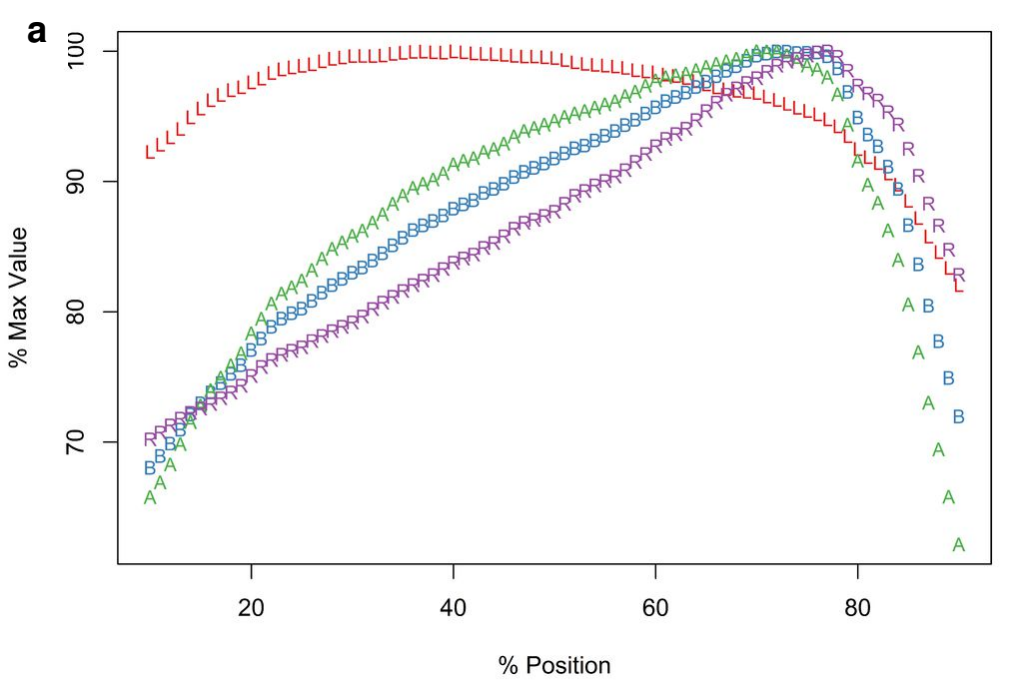
Drummore, southwest Scotland

**Figure 5b.**
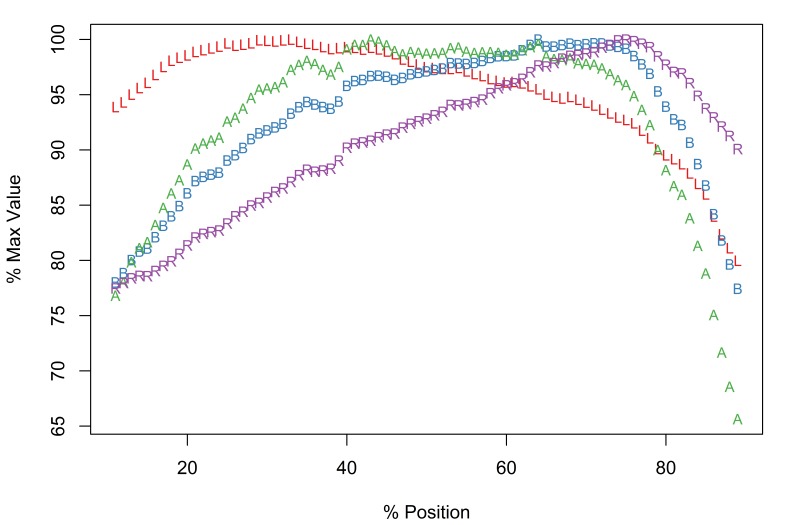
River Carron catchment, central Scotland

**Figure 6a.**
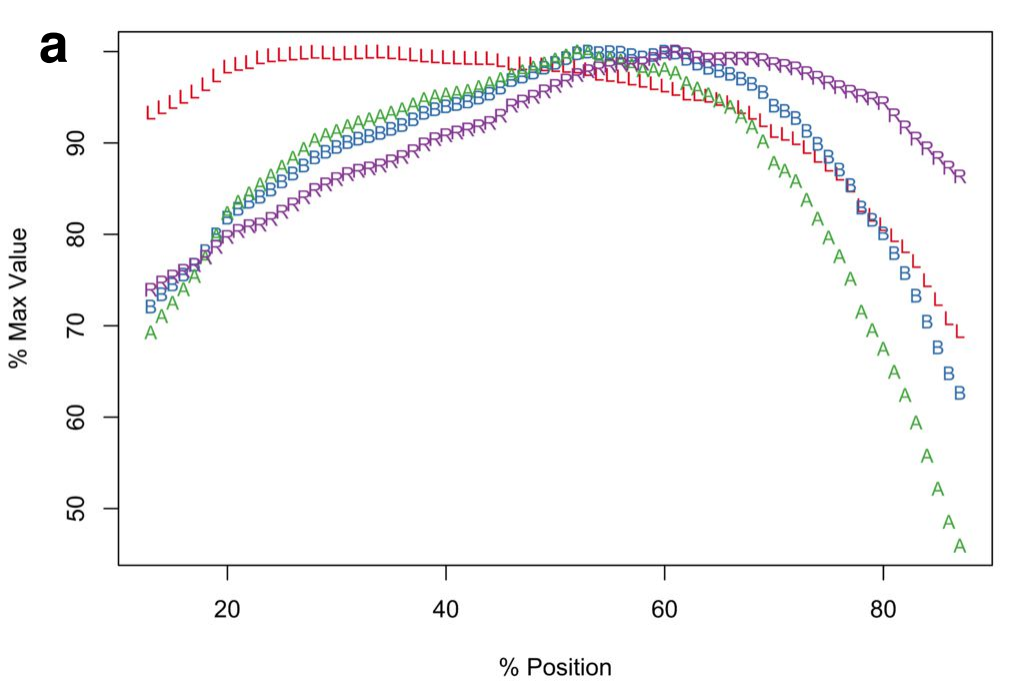
*E. bothniensis*

**Figure 6b.**
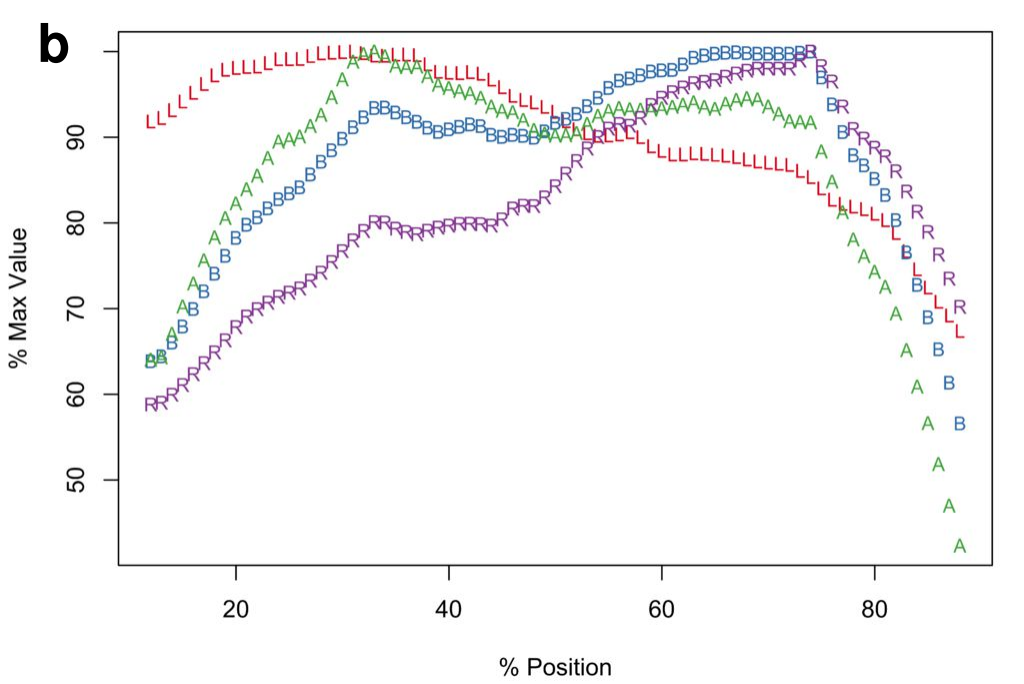
*E. 'bothniensis'*

**Figure 6c.**
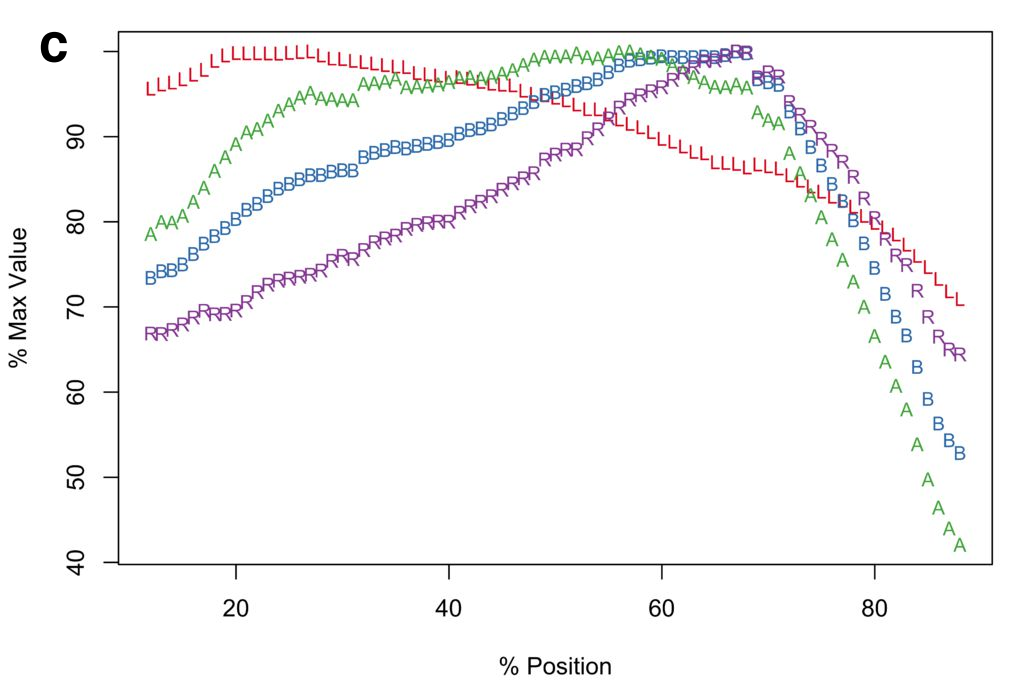
E. gadi sp. A

**Figure 6d.**
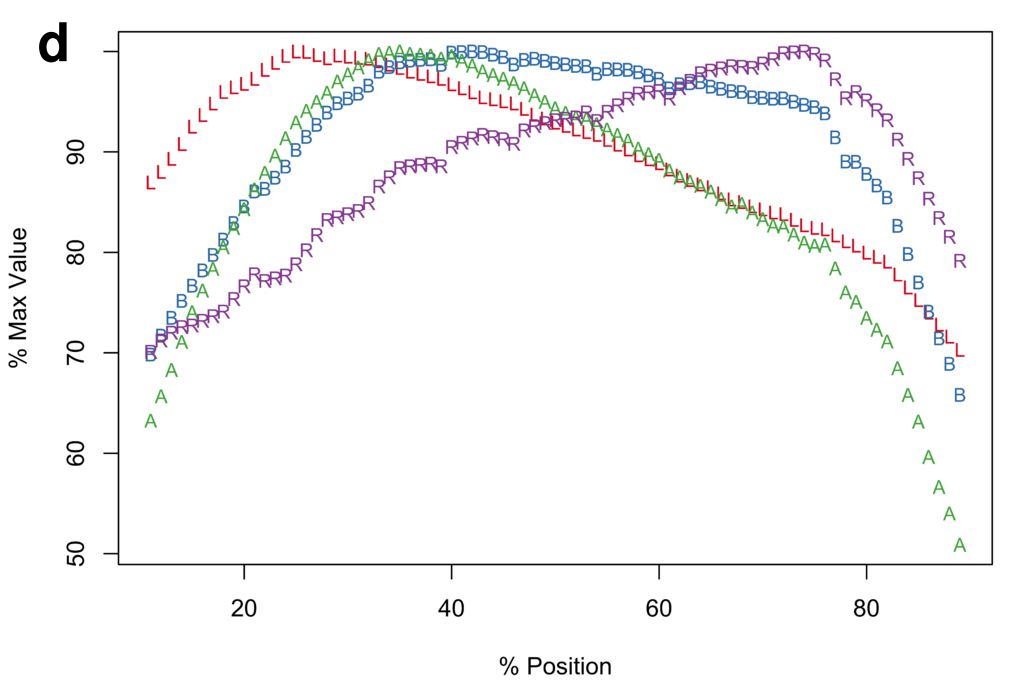
E. gadi sp. B

**Figure 6e.**
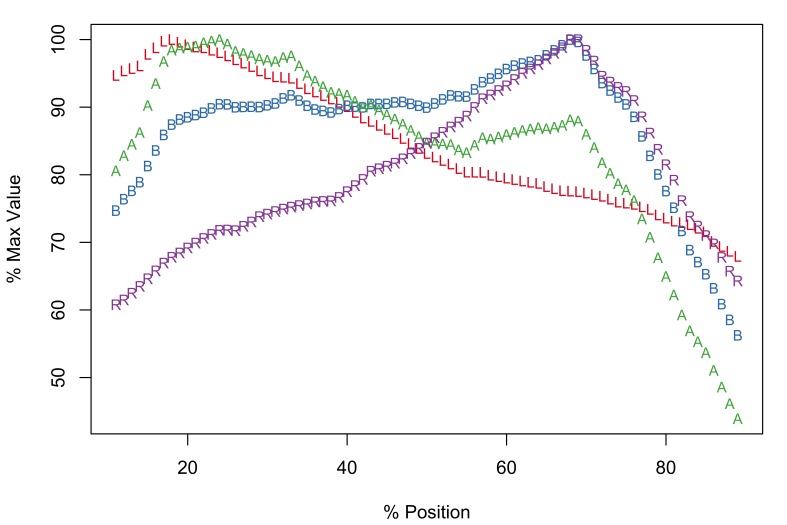
E. gadi sp. I

**Figure 7a.**
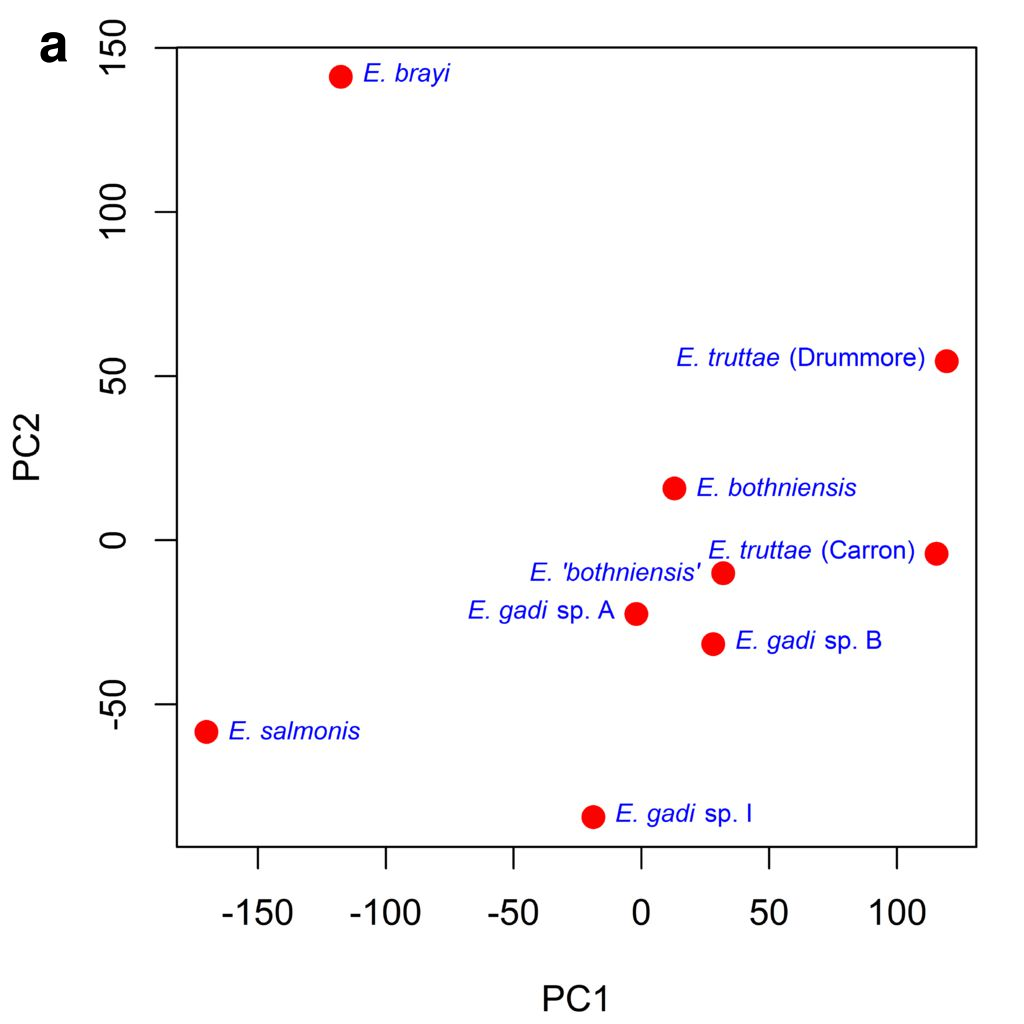
Scatterplot of the scores for the first two principal components (PC1 and PC2).

**Figure 7b.**
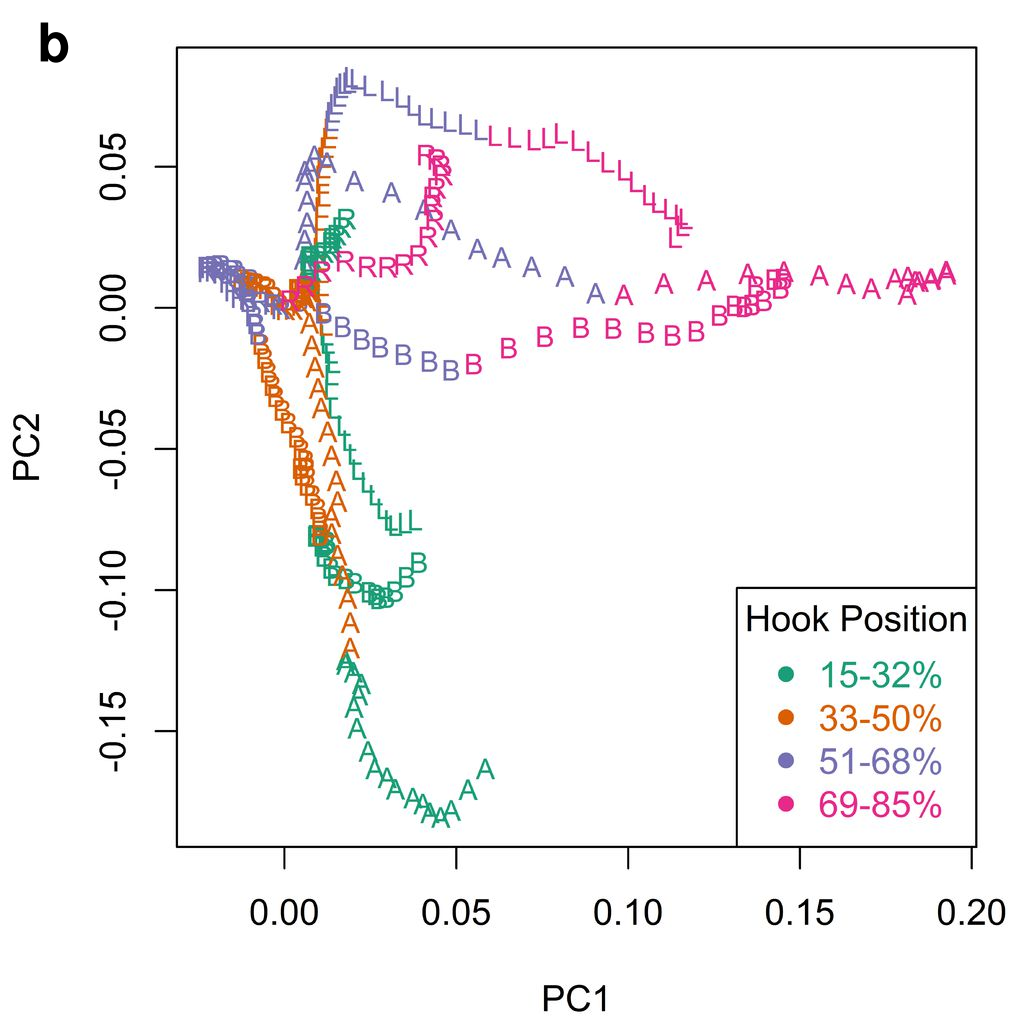
Scatterplot of the loadings for the first two principal components (PC1 and PC2). Hook variables (L, B, A and R) are colour coded to indicate standardized position (%).

**Figure 8a.**
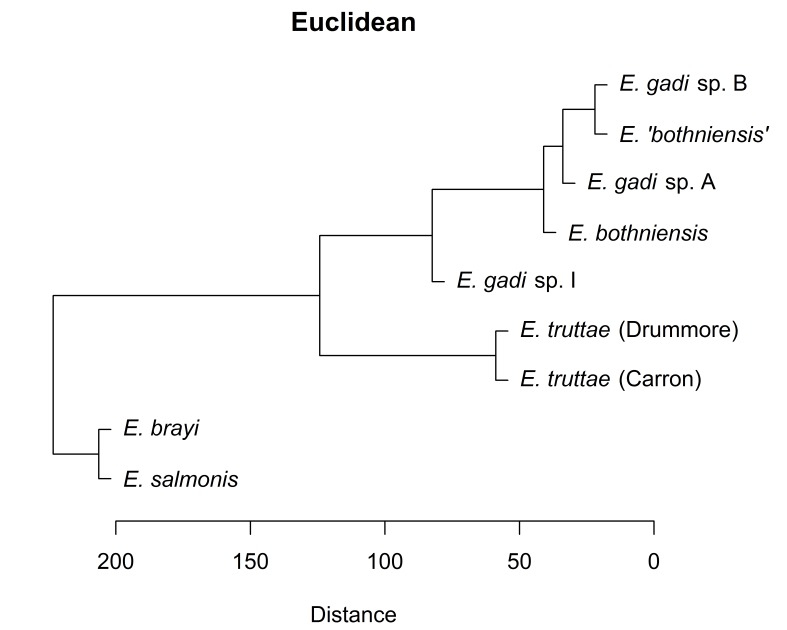
UPGMA clustering using Euclidean distance metric.

**Figure 8b.**
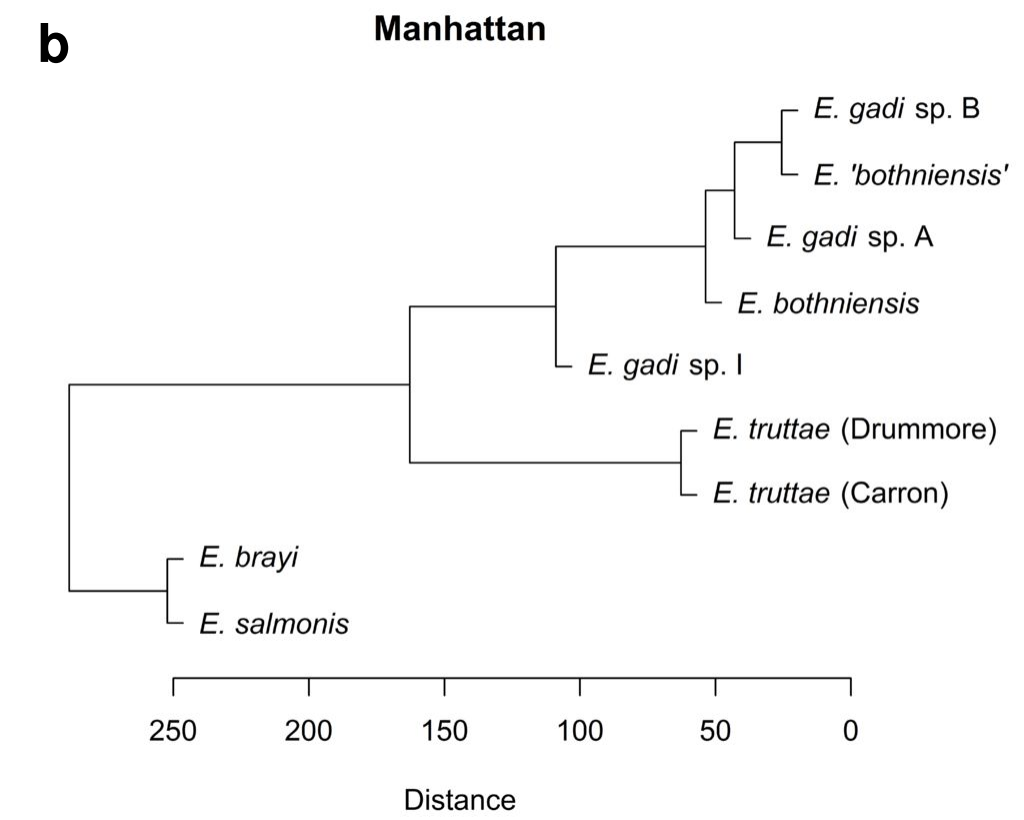
UPGMA clustering using Manhattan distance metric.

**Figure 8c.**
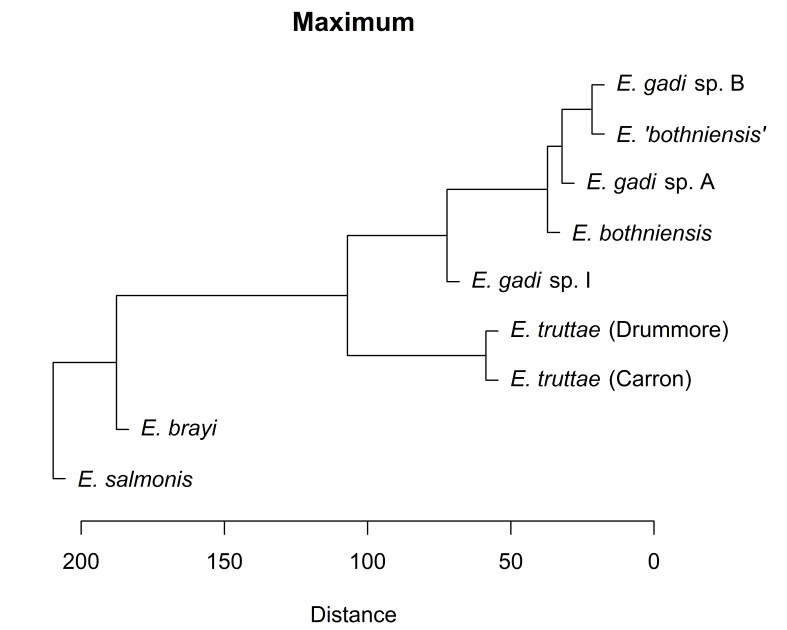
UPGMA clustering using maximum distance metric.

**Figure 8d.**
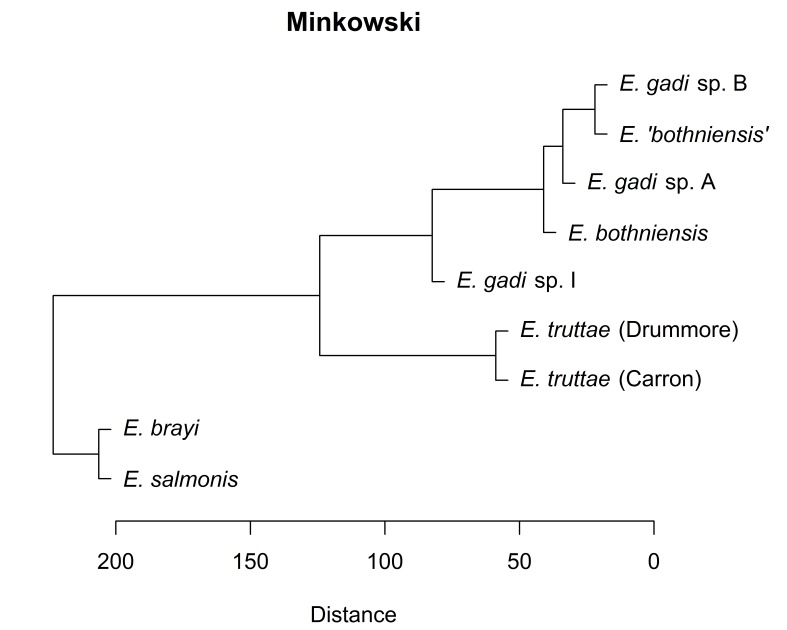
UPGMA clustering using Minkowski distance metric.

**Table 1.** Taxa analysed using the Meristogram. Data files contain hook measurement data in the appropriate format for input to the Meristogram software.

Acanthocephalan	Host(s)	Locality	Accession Numbers	Number of Specimens	Source	Data Files (F, female; M, male)
*E. bothniensis*	*Osmerus eperlanus* L.	Bothnian Bay, Baltic Sea and Lake Keitele, central Finland	BM (NH) 1987.1070–1074 (paratypes); BM (NH) 2002.2.4.102–122; BM (NH) 1989.1474–1491	10 (5 F, 5 M)	Wayland (2013)	F: Suppl. material 3 M: Suppl. material 4
*E. 'bothniensis'*	*Platichthys flesus* (L.)	Lake Pulmankijärvi, northern Finland	NA	2 (2 F, 0 M)	Wayland (2013)	Suppl. material 5
*E. brayi*	*Pachycara crassiceps* (Roule)	Porcupine Seabight, 49°49.9'N, 13°08.2'W, depth 2,444 m	BM(NH) 1997.12.8.3 (holotype); BM(NH) 1997.12.8.4–28	11 (4 F, 7 M)	Wayland et al. (2013c)	F: Suppl. material 6 M: Suppl. material 7
*E. gadi* sp. A	*Gadus morhua* L.	northern North Sea	NA	6 (4 F, 2 M)	Wayland et al. (2013b)	F: Suppl. material 8 M: Suppl. material 9
*E. gadi* sp. B	*G. morhua* and *Melanogrammus aeglefinus* (L.)	northern North Sea	NA	8 (4 F, 4 M)	Wayland et al. (2013b)	F: Suppl. material 10 M: Suppl. material 11
*E. gadi* sp. I	*G. morhua*	Baltic Sea, off Tvärminne, Hanko	BM(NH) 2002.2.4.90–101	6 (5 F, 1 M)	Wayland et al. (2013b)	F: Suppl. material 12 M: Suppl. material 13
*E. salmonis*	*Coregonus lavaretus* L. and *Osmerus eperlanus* (L.)	Bothnian Bay, Baltic Sea	BMNH 2002.2.4.132-226; BMNH 2002.2.4.227-263	42 (36 F, 6 M)	Wayland et al. (2013a)	F dorsal: Suppl. material 14 F ventral: Suppl. material 15 M dorsal: Suppl. material 16 M ventral: Suppl. material 17
*E. truttae*	*Salmo trutta* L.	Drummore, southwest Scotland	BM (NH) 1986.764–793	54 (35 F, 19 M)	Wayland 2013	F: Suppl. material 18 M: Suppl. material 19
*E. truttae*	*S. trutta*	River Carron catchment, central Scotland	BM (NH) 2002.2.4.264–275; BM (NH) 2002.2.4.276–283	18 (11 F, 7 M)	Wayland (2013)	F: Suppl. material 20 M: Suppl. material 21
