# Supplementary figures and images for: The Meristogram: a neglected tool for acanthocephalan systematics
Source: Biodivers Data J. 2016 Feb 4;(4):e7606. doi: 10.3897/BDJ.4.e7606 (PMC4759448; doi:10.3897/BDJ.4.e7606)

**female 9%**

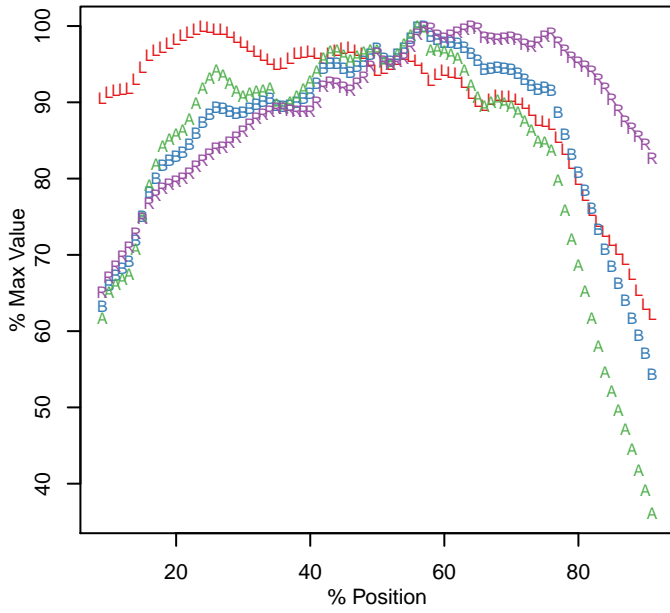

**male 9%**

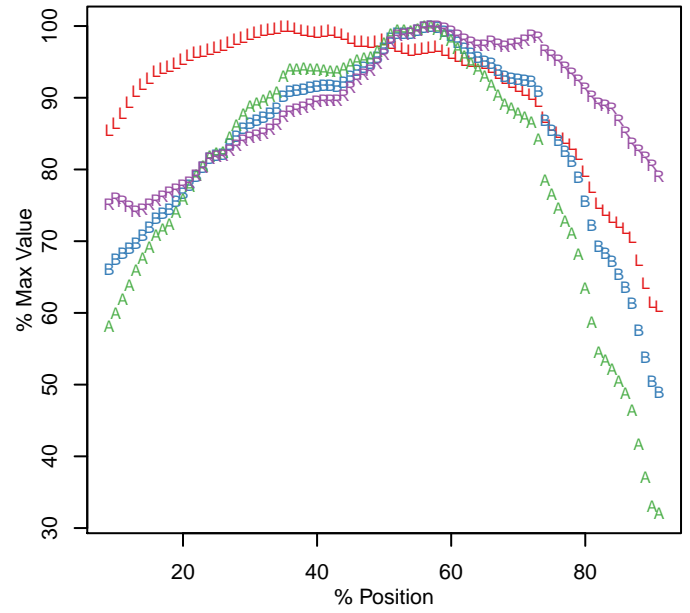

**female 14%**

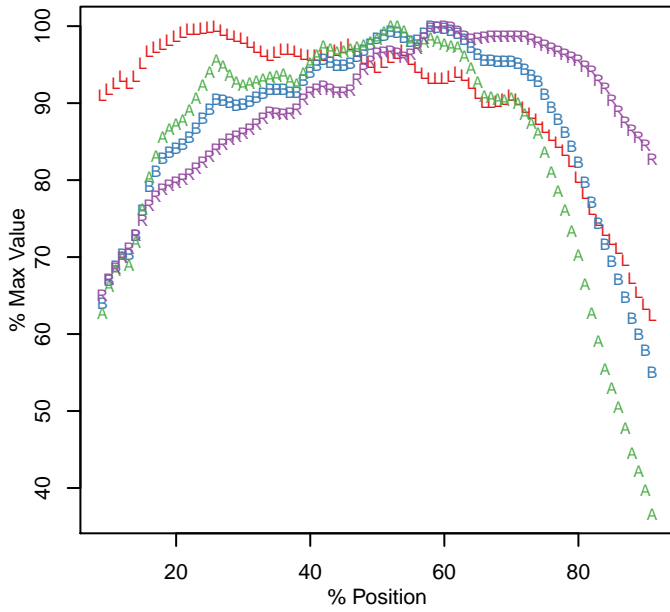

**male 14%**

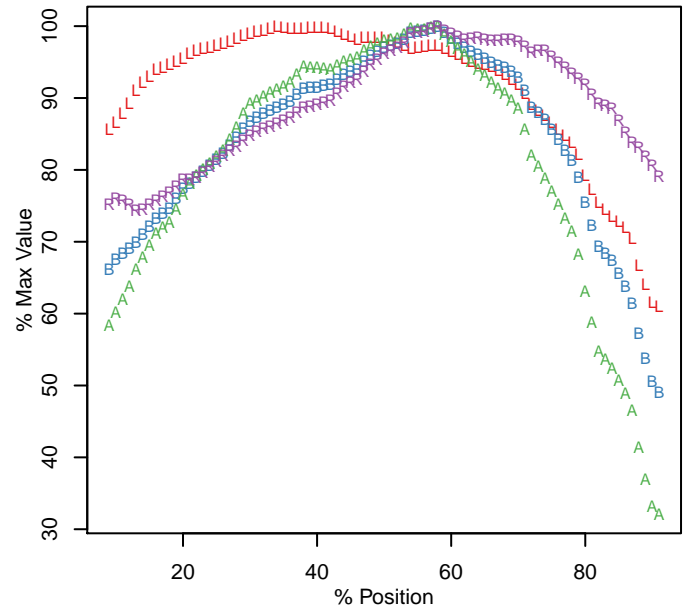

**female 18%**

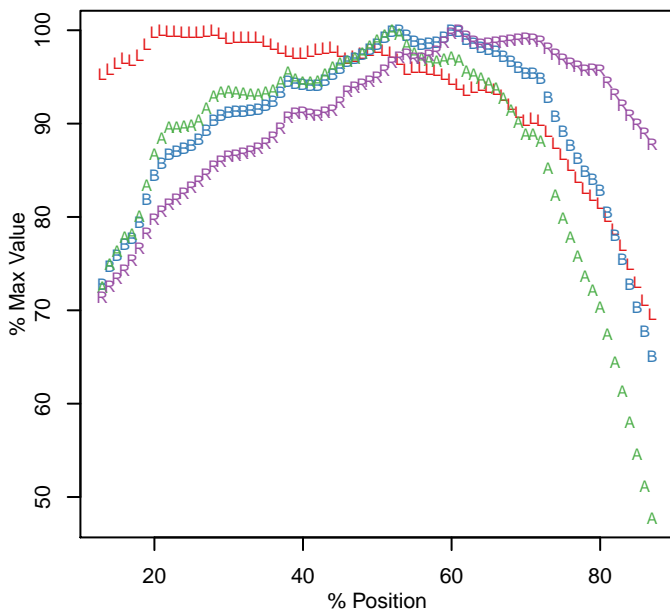

**male 18%**

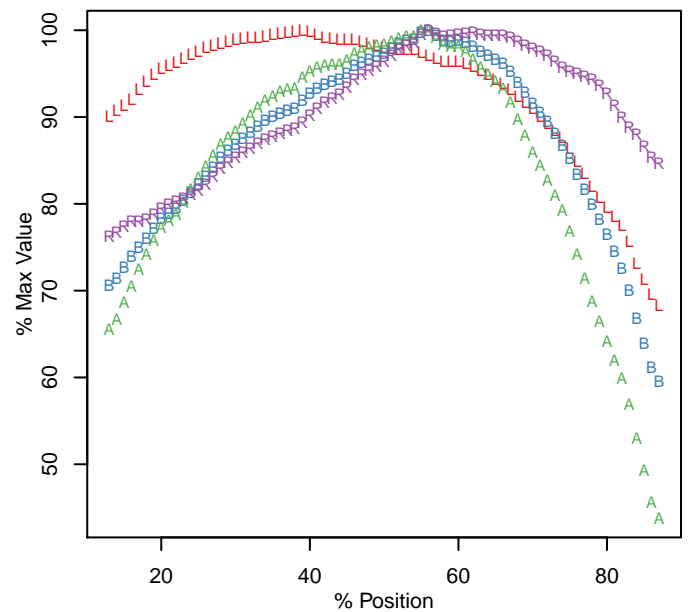

Supplement: Supplementary material 22 — Meristograms for female and male Echinorhynchus bothniensis [file biodiversity_data_journal-4-e7606-s022.pdf]

**female 8%**

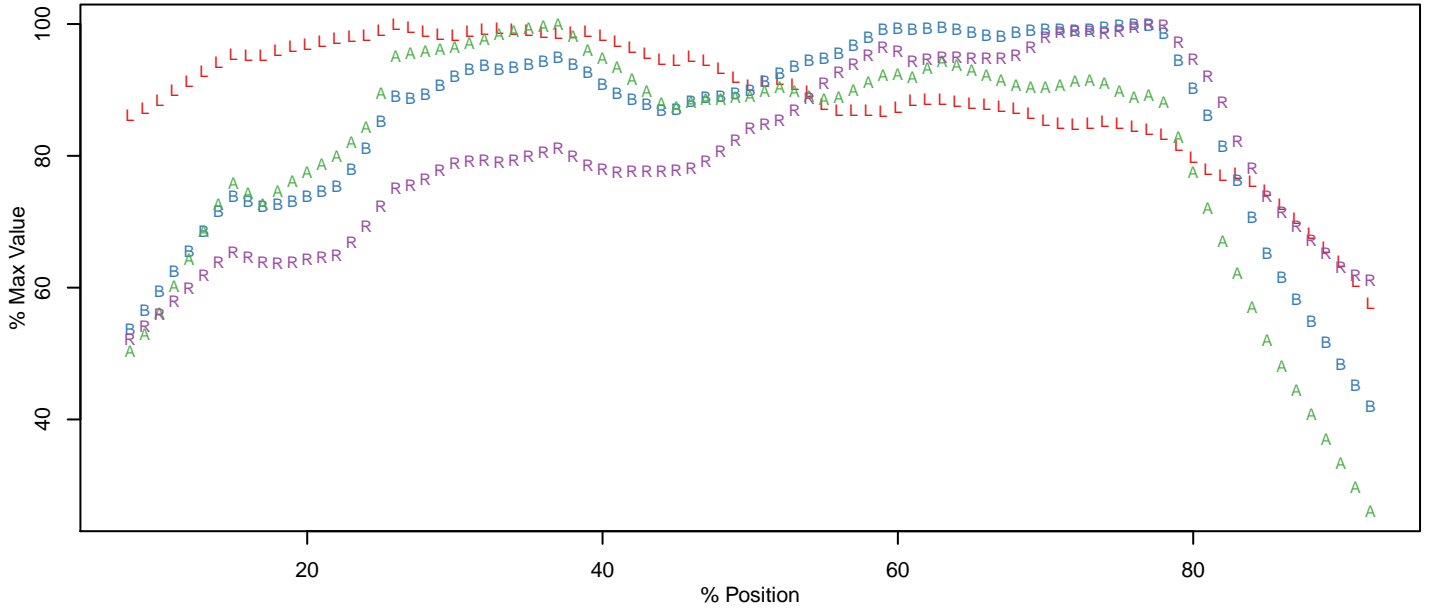

**female 12%**

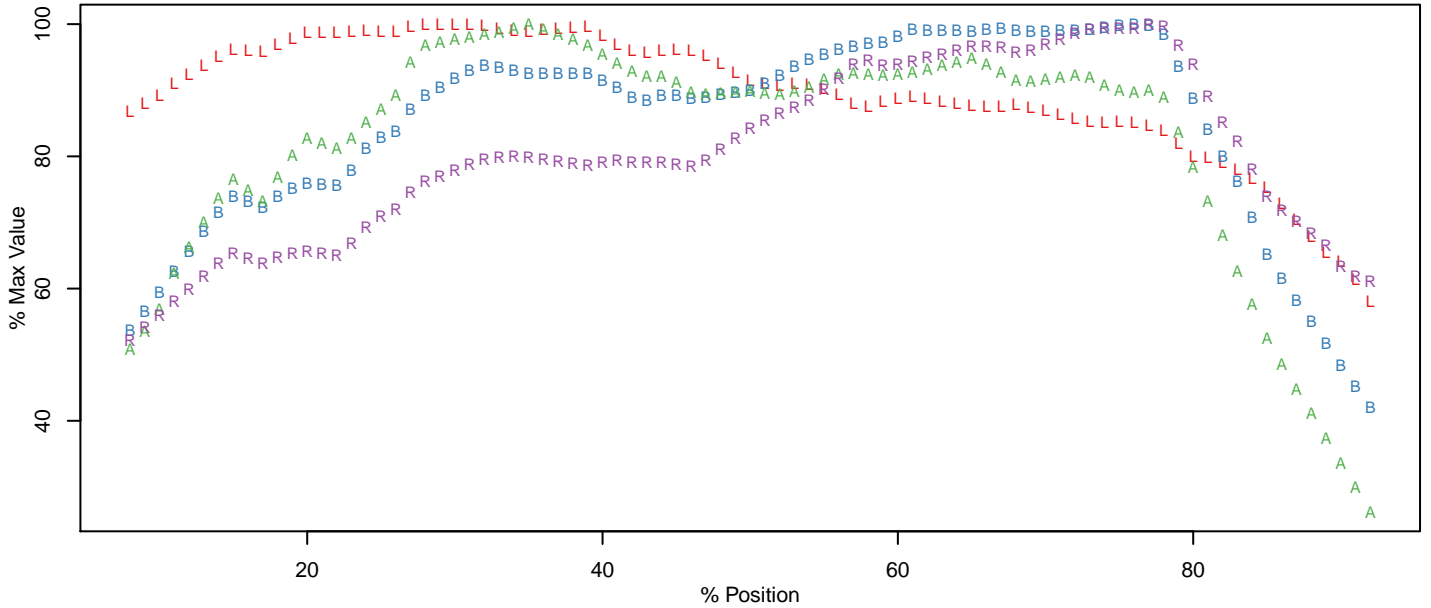

**female 16%**

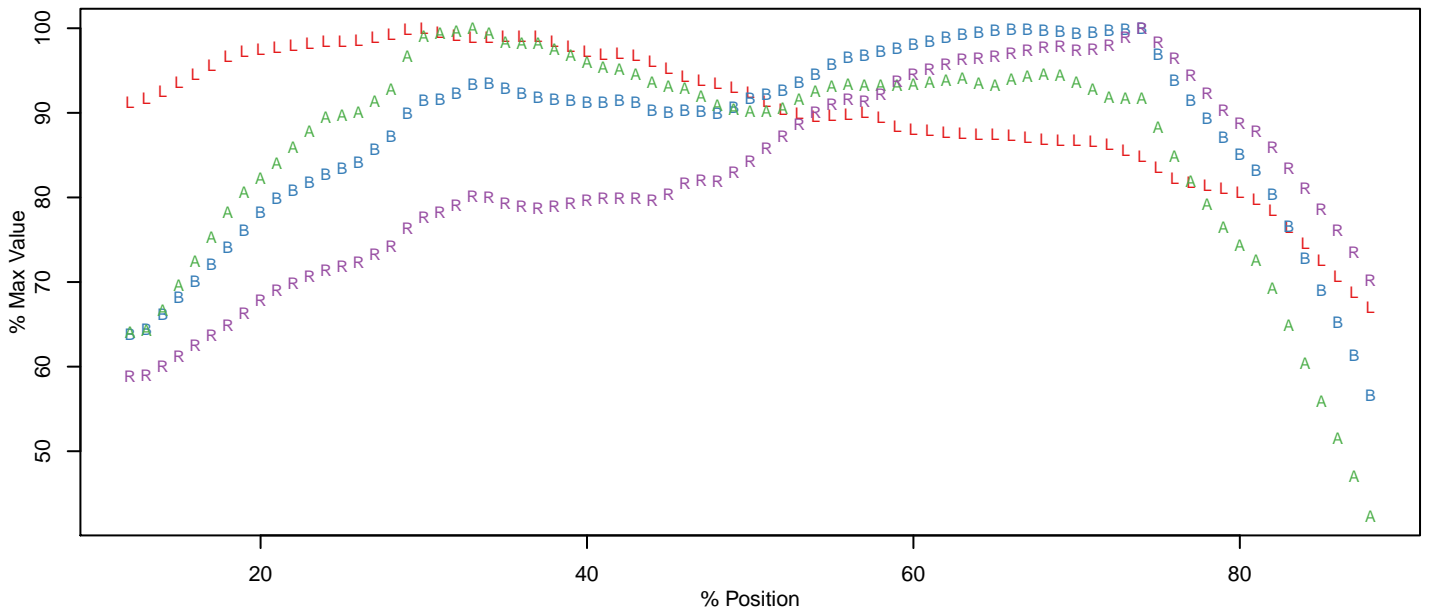

Supplement: Supplementary material 23 — Meristograms for female Echinorhynchus 'bothniensis' [file biodiversity_data_journal-4-e7606-s023.pdf]

**female 17%**

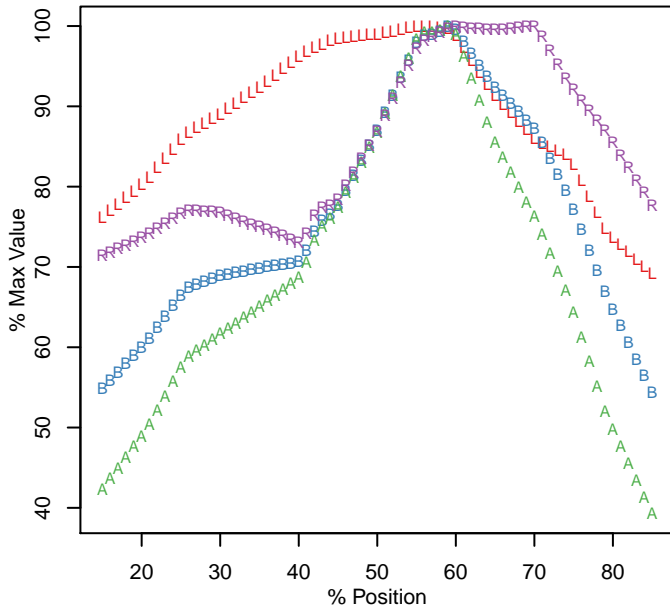

**male 17%**

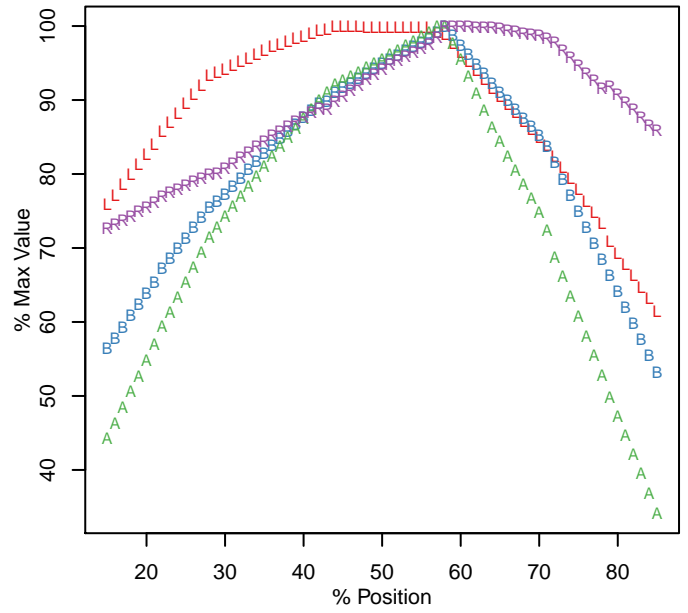

**female 26%**

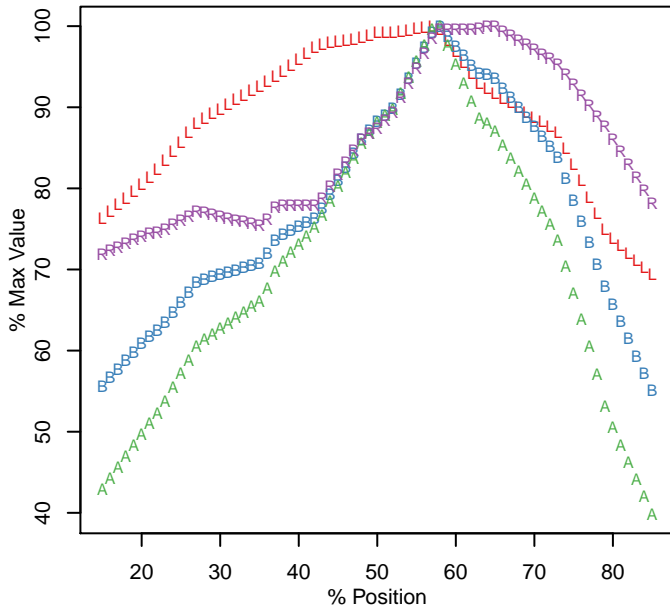

**male 26%**

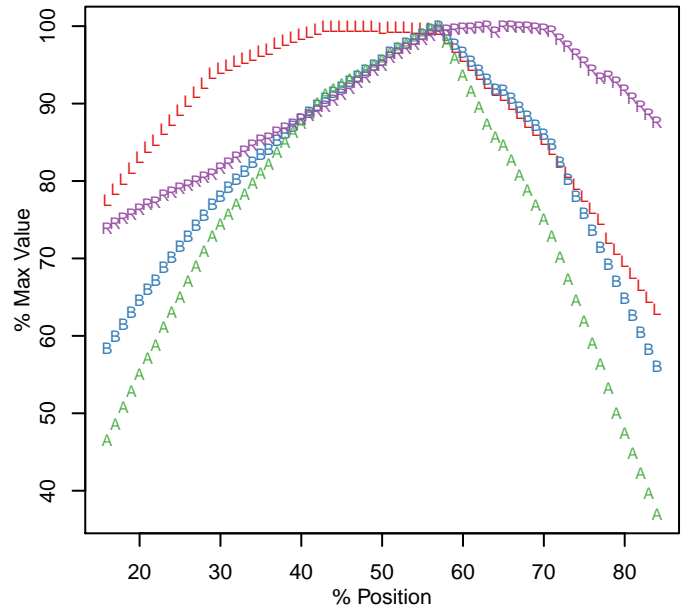

**female 34%**

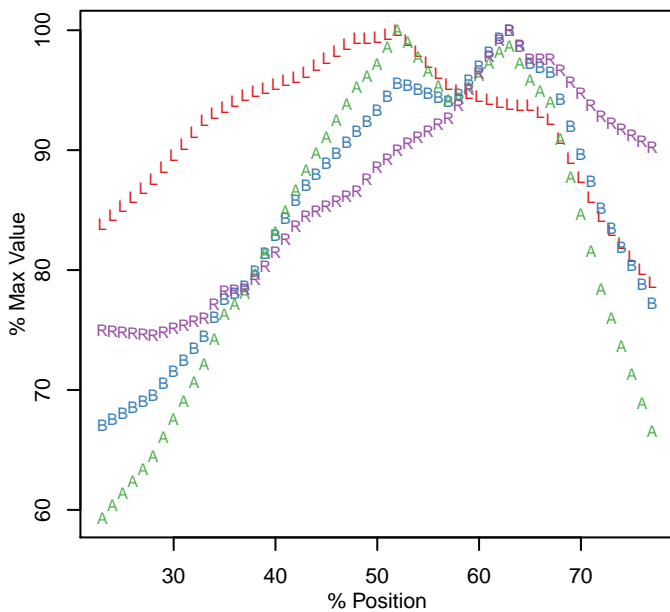

**male 34%**

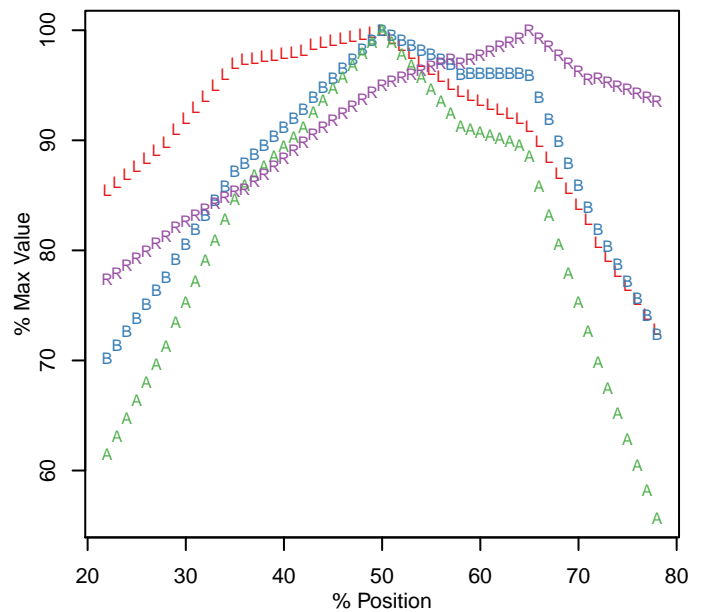

Supplement: Supplementary material 24 — Meristograms for female and male Echinorhynchus brayi [file biodiversity_data_journal-4-e7606-s024.pdf]

**female 9%**

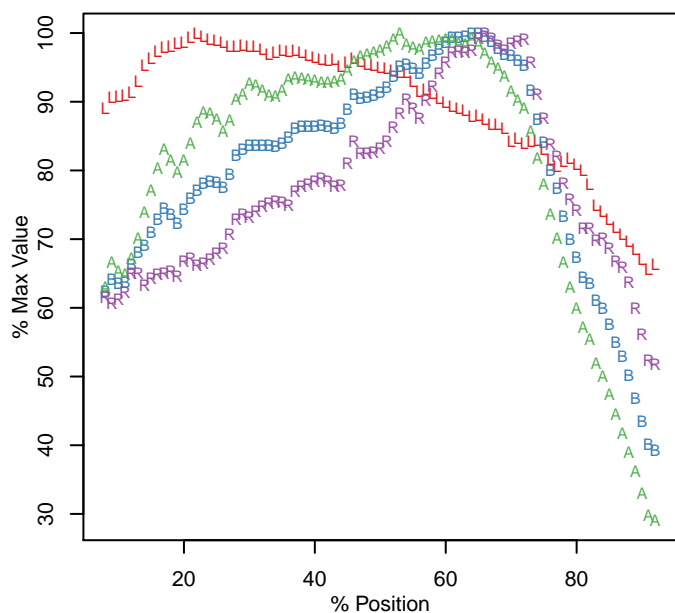

**male 9%**

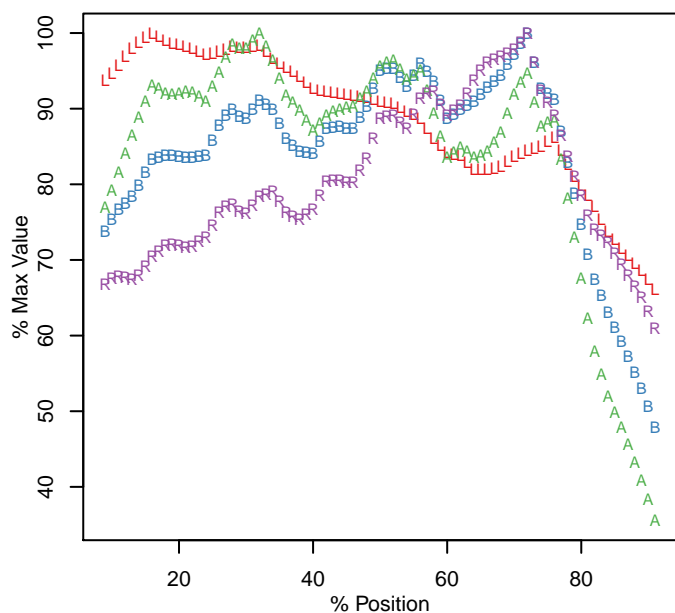

**female 14%**

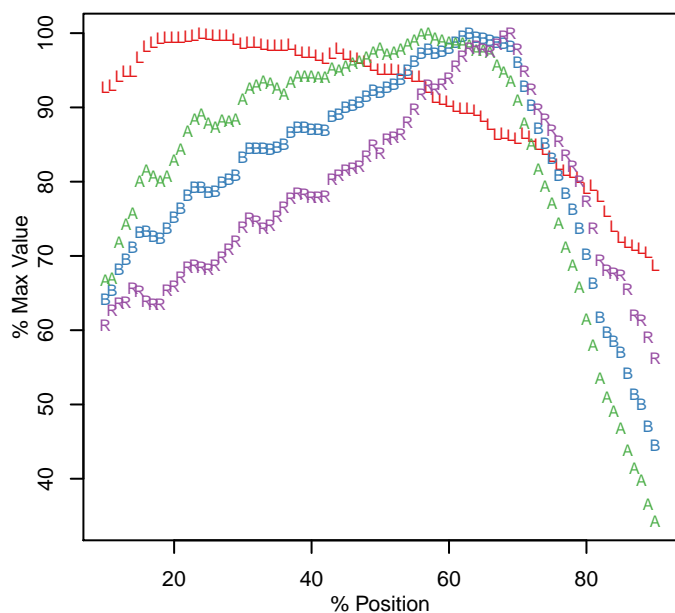

**male 14%**

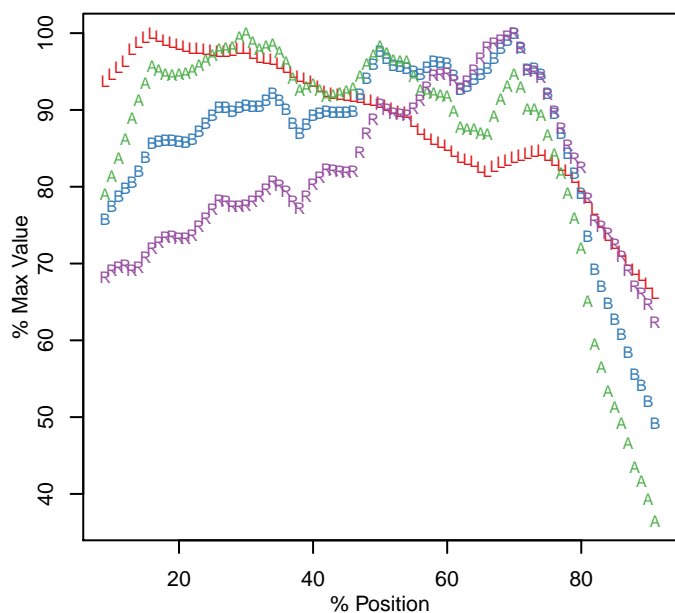

**female 18%**

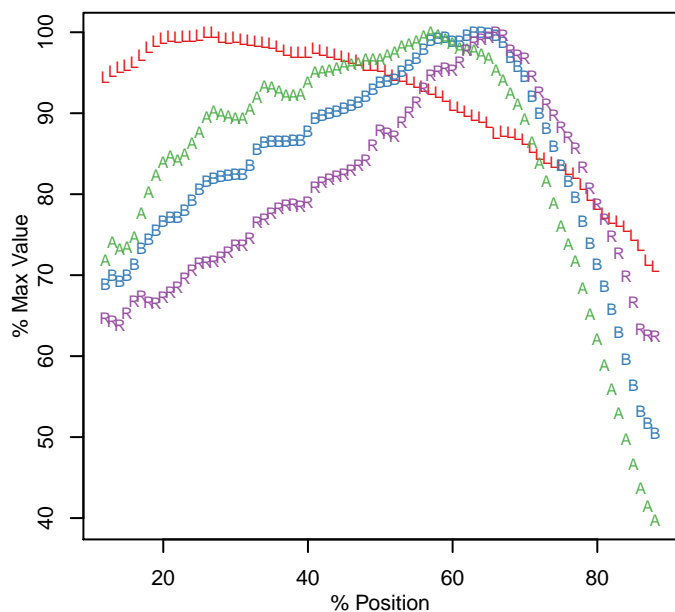

**male 18%**

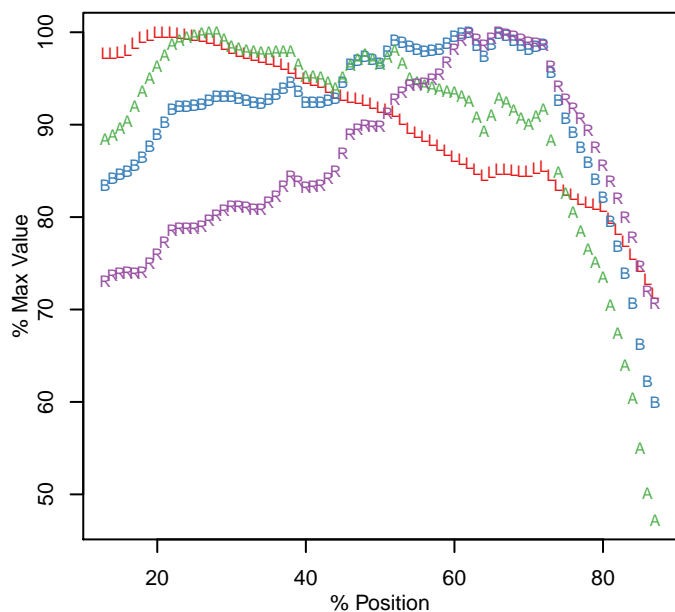

Supplement: Supplementary material 25 — Meristograms for female and male Echinorhynchus gadi sp. A [file biodiversity_data_journal-4-e7606-s025.pdf]

**female 8%**

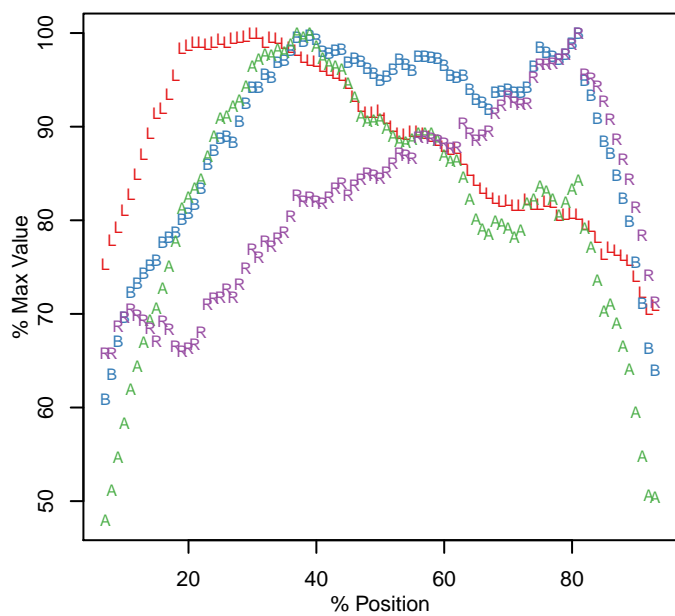

**male 8%**

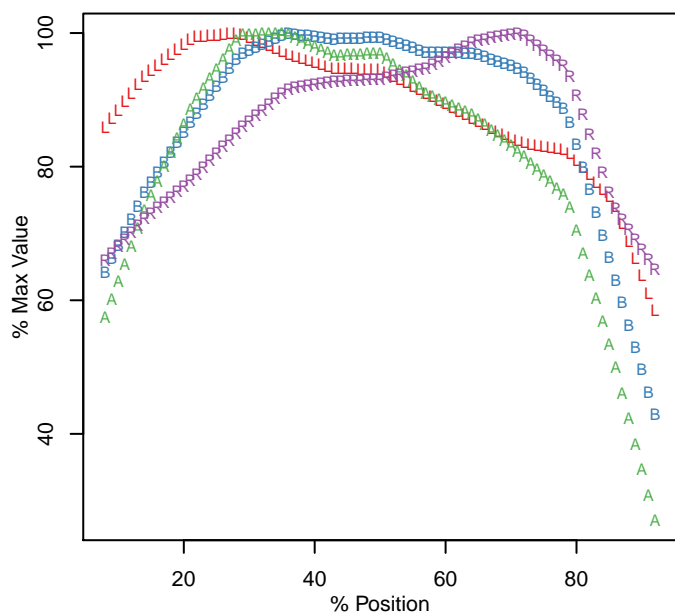

**female 12%**

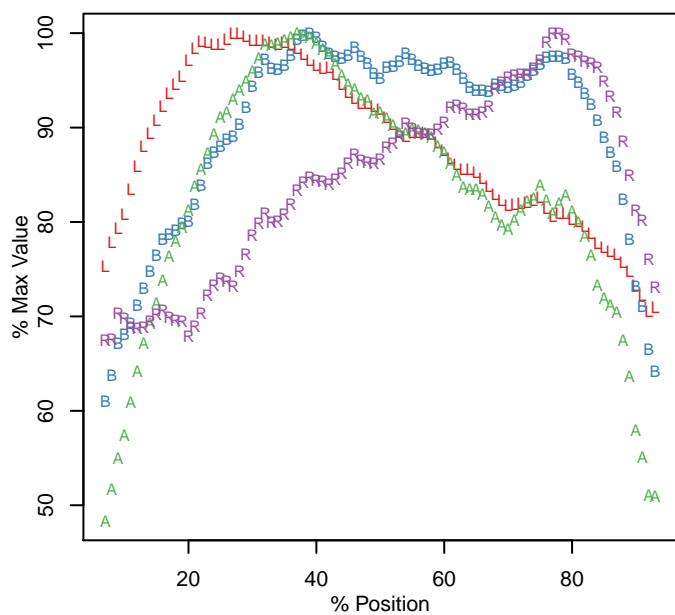

**male 12%**

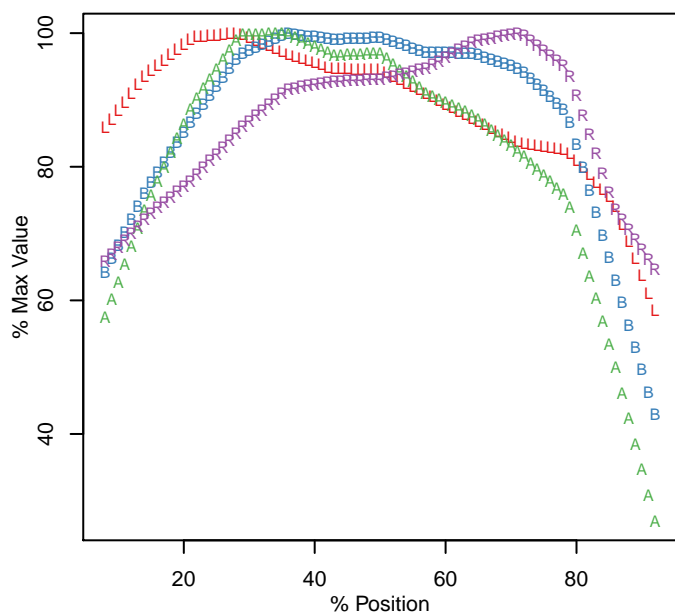

**female 16%**

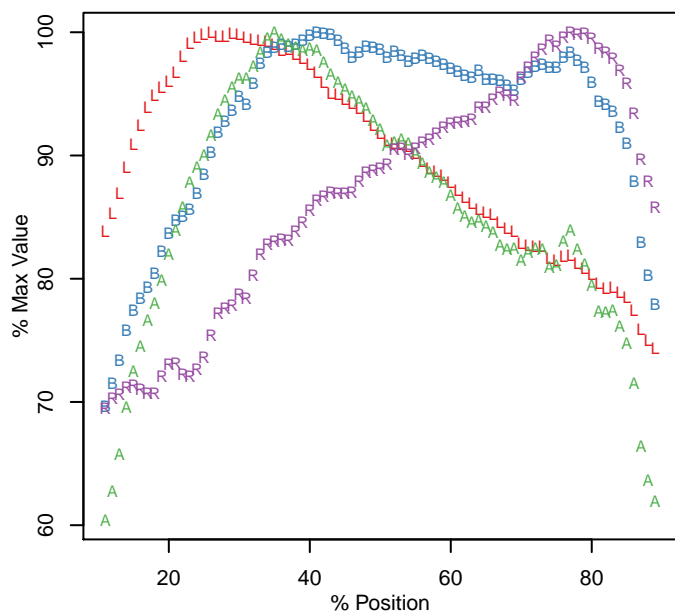

**male 16%**

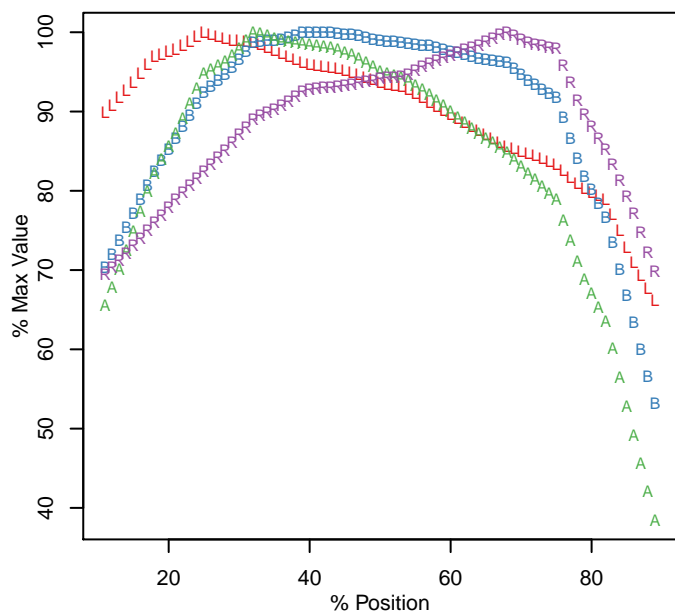

Supplement: Supplementary material 26 — Meristograms for female and male Echinorhynchus gadi sp. B [file biodiversity_data_journal-4-e7606-s026.pdf]

**female 8%**

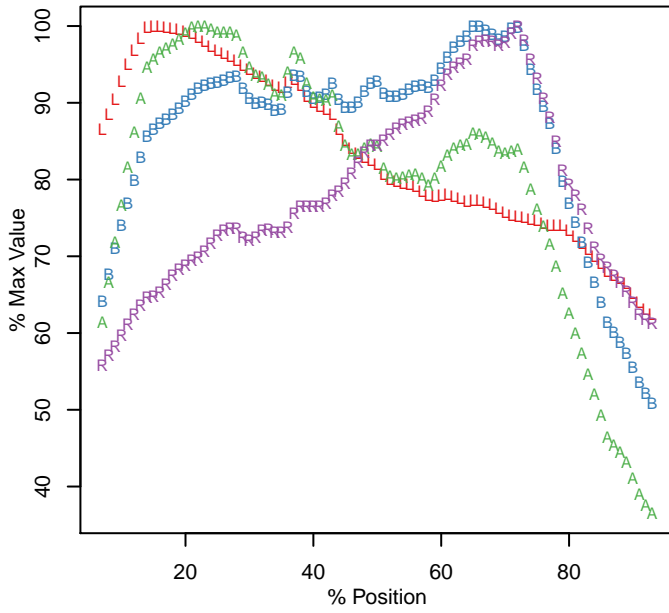

**male 8%**

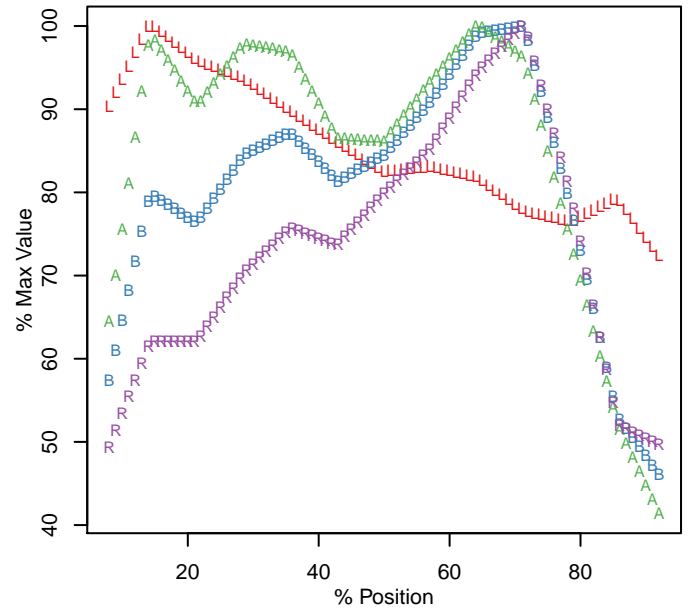

**female 12%**

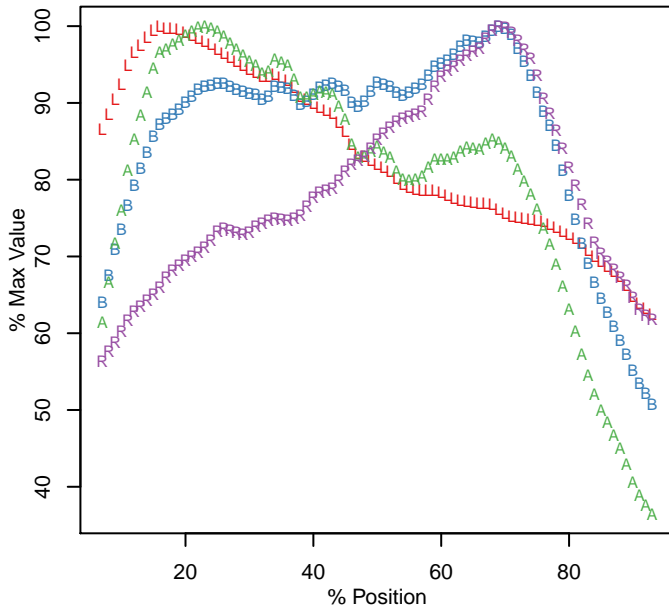

**male 12%**

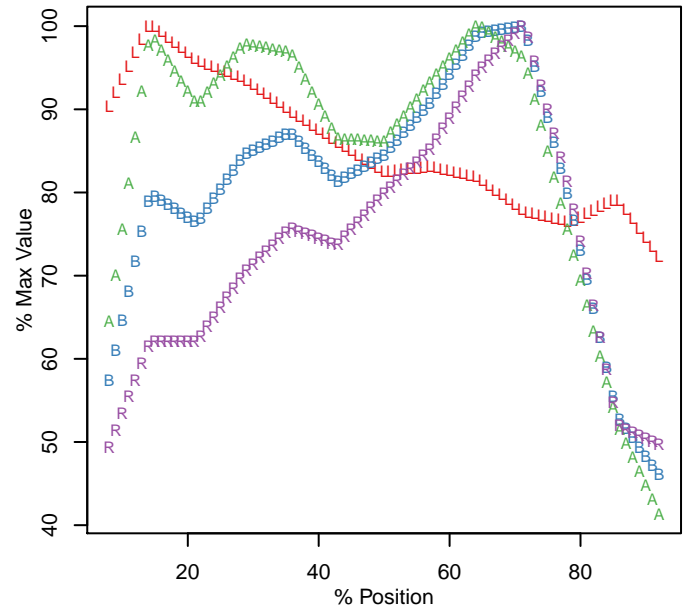

**female 16%**

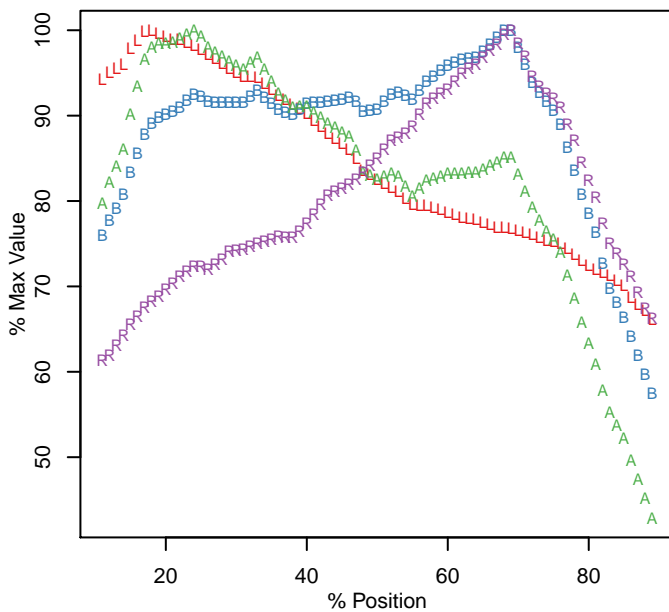

**male 16%**

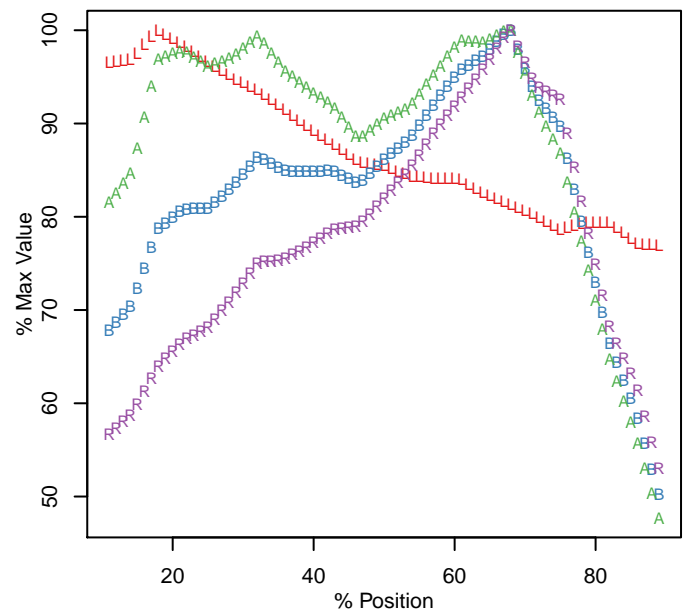

Supplement: Supplementary material 27 — Meristograms for female and male Echinorhynchus gadi sp. I [file biodiversity_data_journal-4-e7606-s027.pdf]
