# Supplementary material for: The Meristogram: a neglected tool for acanthocephalan systematics
Source: Biodivers Data J. 2016 Feb 4;(4):e7606. doi: 10.3897/BDJ.4.e7606 (PMC4759448; doi:10.3897/BDJ.4.e7606)
Supplement: Supplementary material 28 — Meristograms for female and male Echinorhynchus salmonis [file biodiversity_data_journal-4-e7606-s028.pdf]

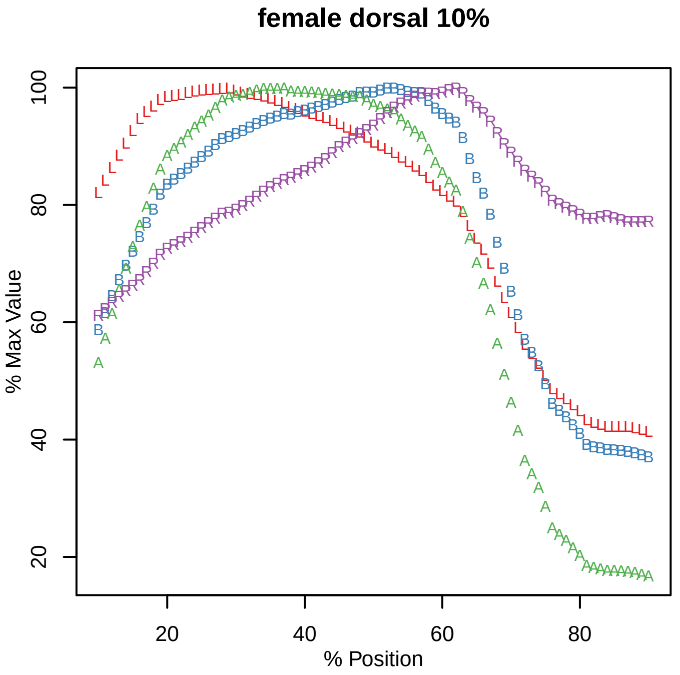
female dorsal 10%
% Max Value
20
40
60
80
100
20
40
60
80
% Position

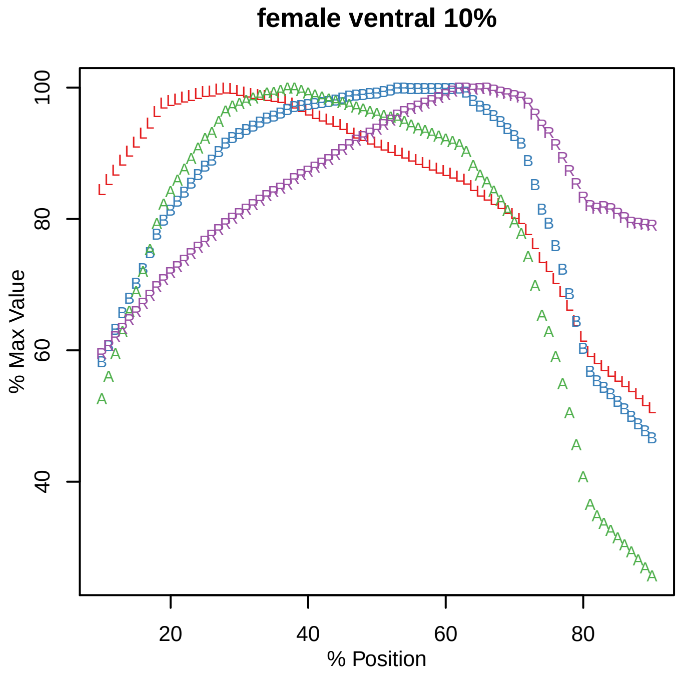
female ventral 10%
% Max Value
40
60
80
100
20
40
60
80
% Position

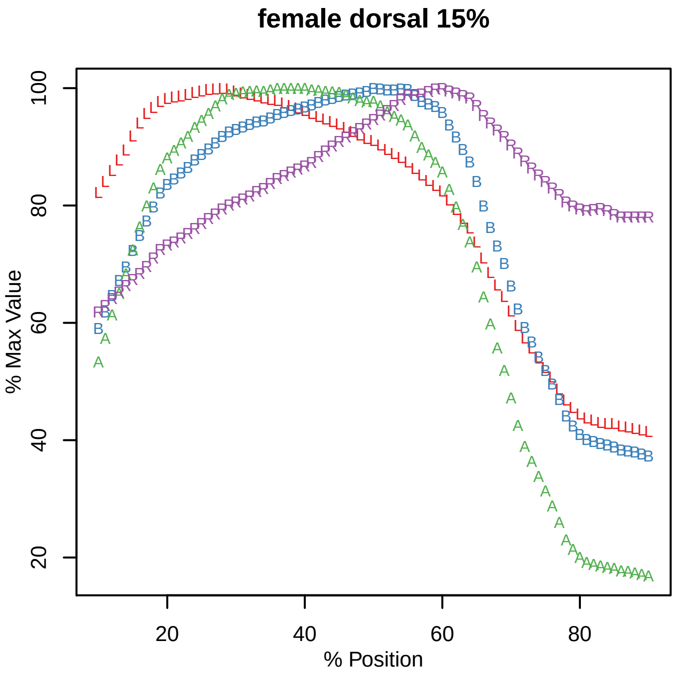
female dorsal 15%
% Max Value
20
40
60
80
100
20
40
60
80
% Position

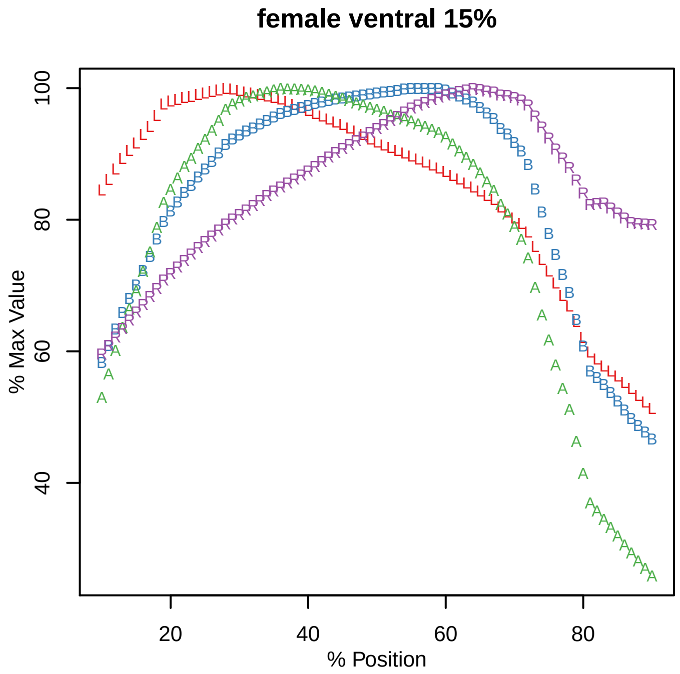
female ventral 15%
% Max Value
40
60
80
100
20
40
60
80
% Position

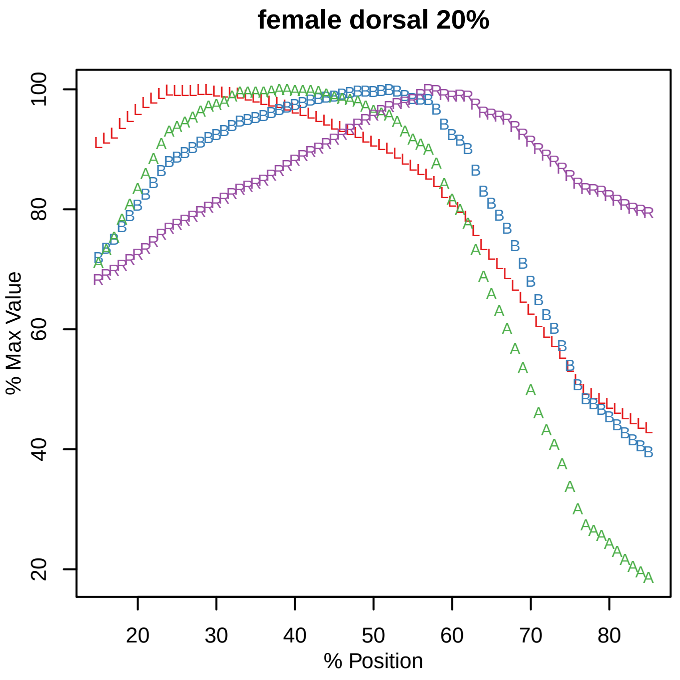
female dorsal 20%
% Max Value
20
40
60
80
100
20
30
40
50
60
70
80
% Position

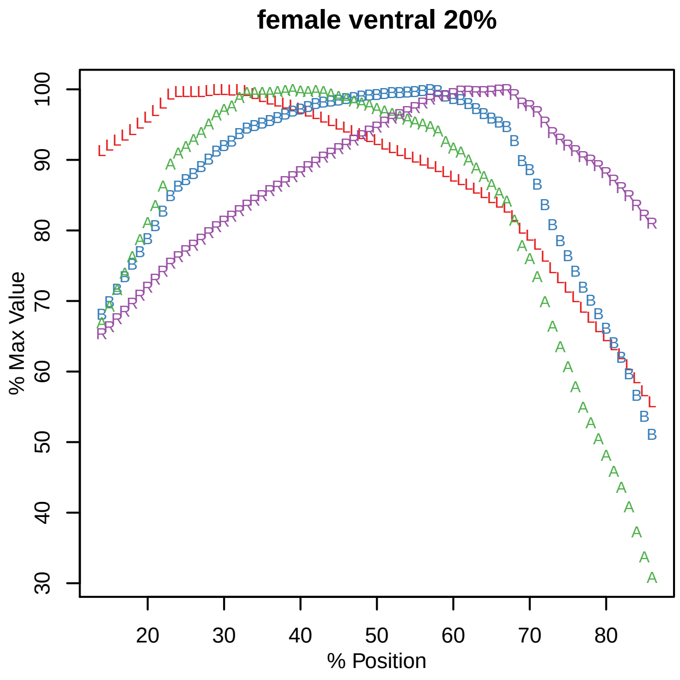
female ventral 20%
% Max Value
30
40
50
60
70
80
90
100
20
30
40
50
60
70
80
% Position

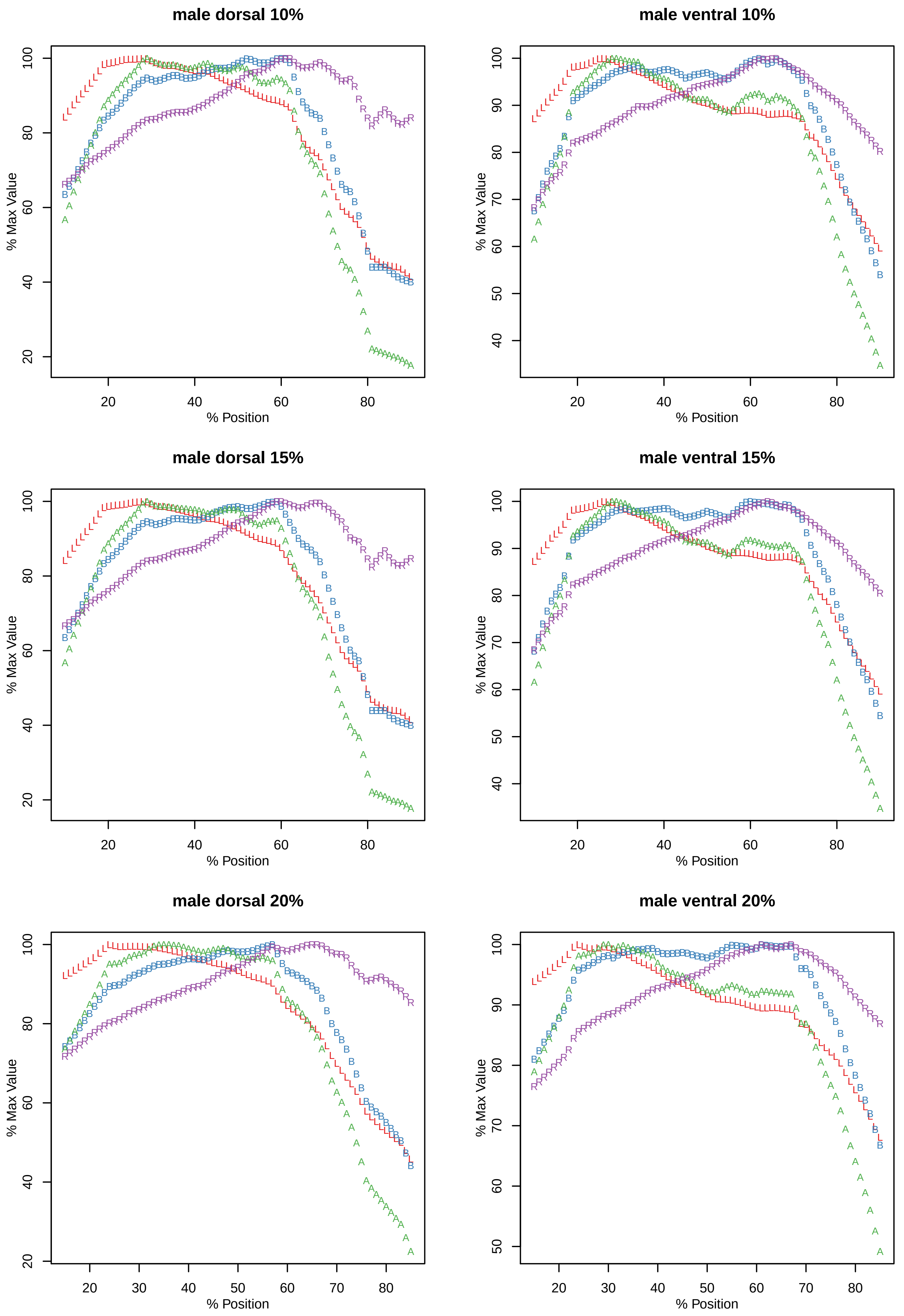
male dorsal 10%
male ventral 10%
male dorsal 15%
male ventral 15%
male dorsal 20%
male ventral 20%
% Max Value
% Position
