# Supplementary material for: The Meristogram: a neglected tool for acanthocephalan systematics
Source: Biodivers Data J. 2016 Feb 4;(4):e7606. doi: 10.3897/BDJ.4.e7606 (PMC4759448; doi:10.3897/BDJ.4.e7606)
Supplement: Supplementary material 29 — Meristograms for female and male Echinorhynchus truttae [file biodiversity_data_journal-4-e7606-s029.pdf]

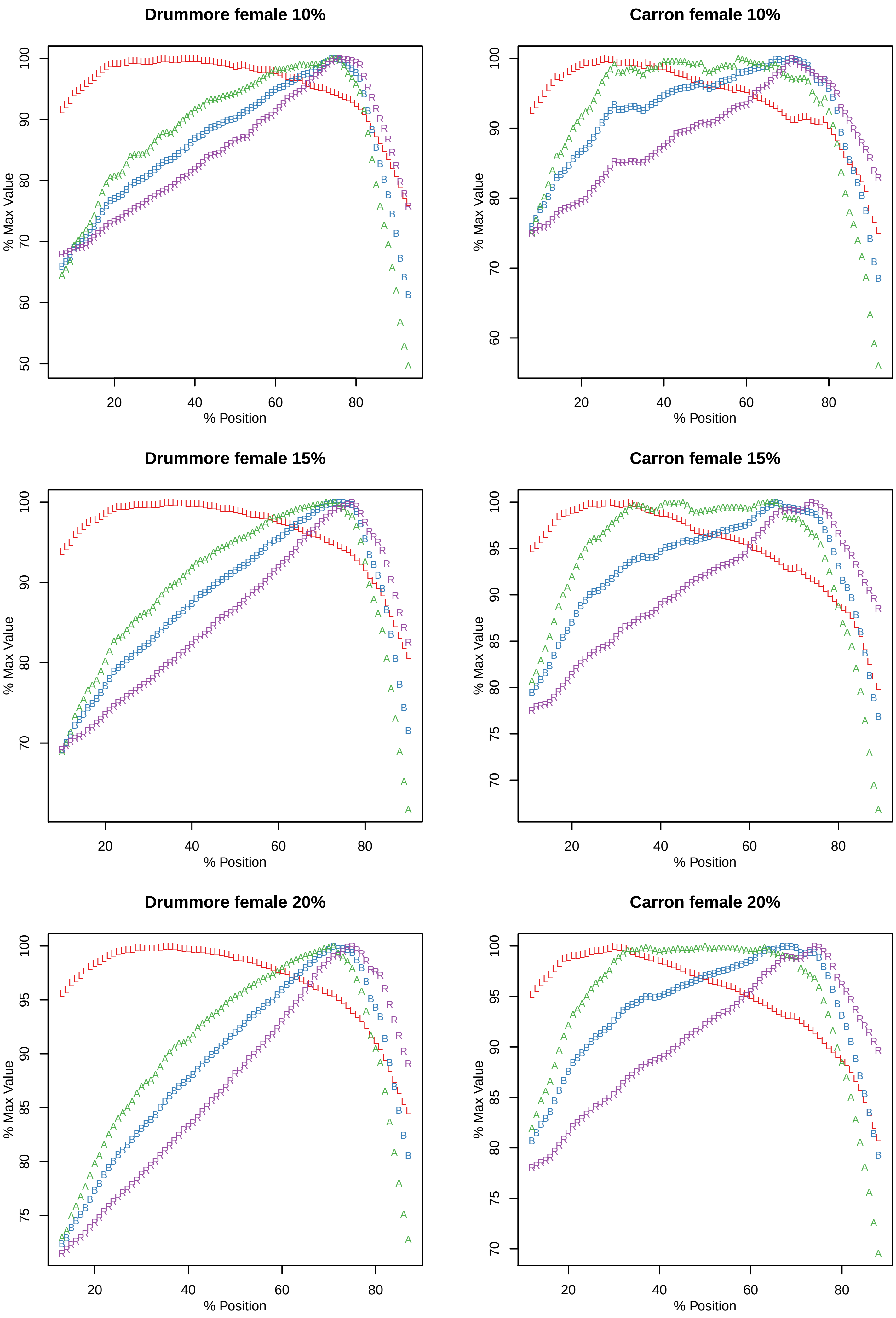
Drummore female 10%
Carron female 10%
Drummore female 15%
Carron female 15%
Drummore female 20%
Carron female 20%
% Max Value
% Position
20
40
60
80
50
60
70
75
80
85
90
95
100

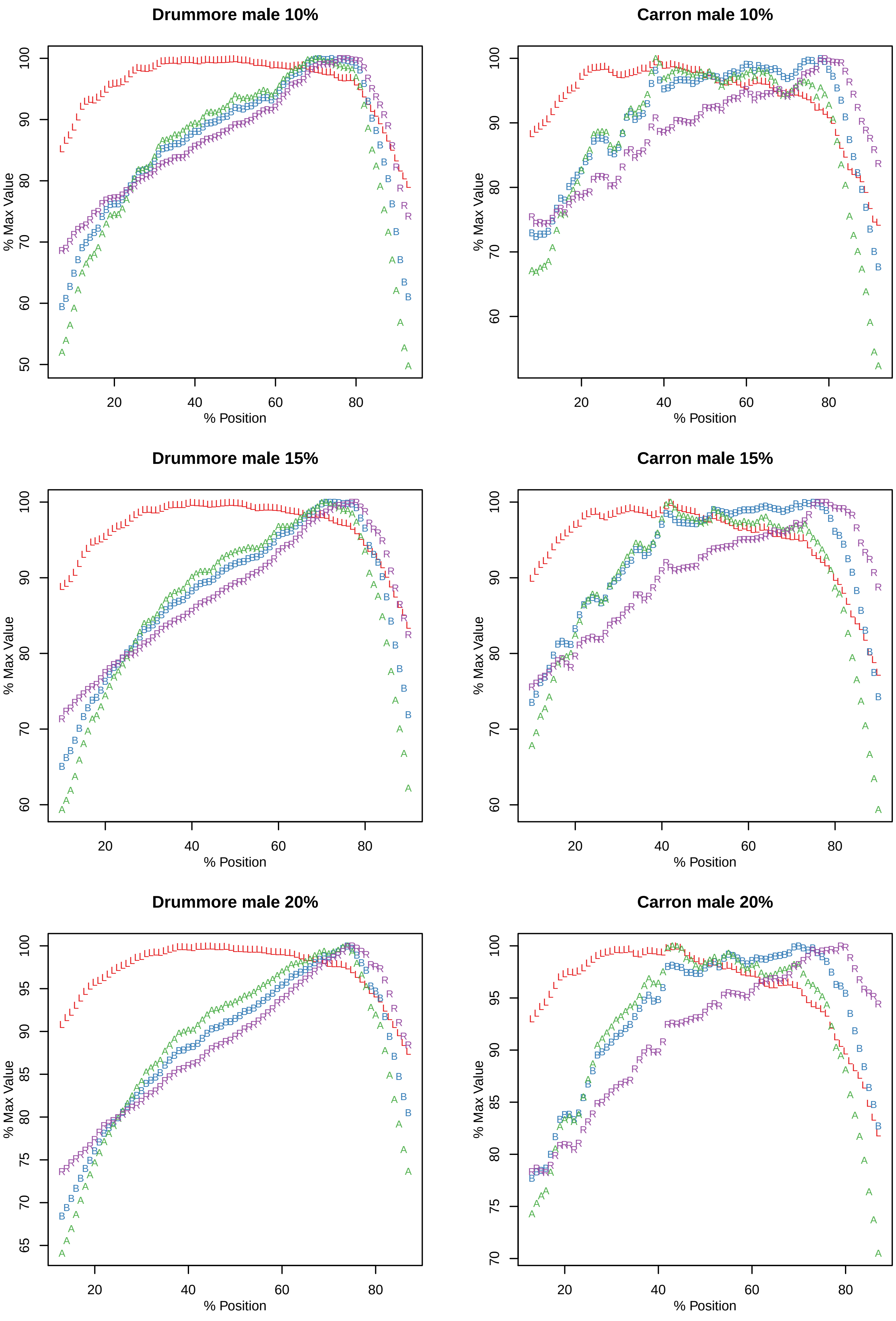
Drummore male 10%
Carron male 10%
Drummore male 15%
Carron male 15%
Drummore male 20%
Carron male 20%
% Max Value
% Position
L
R
B
A
